# Development and validation of QDiabetes-2018 risk prediction algorithm to estimate future risk of type 2 diabetes: cohort study

**DOI:** 10.1136/bmj.j5019

**Published:** 2017-11-20

**Authors:** Julia Hippisley-Cox, Carol Coupland

**Affiliations:** 1 Division of Primary Care, University of Nottingham, Nottingham NG2 7RD, UK; 2 ClinRisk, Leeds, West Yorkshire, UK

## Abstract

**Objectives** To derive and validate updated QDiabetes-2018 prediction algorithms to estimate the 10 year risk of type 2 diabetes in men and women, taking account of potential new risk factors, and to compare their performance with current approaches.

**Design** Prospective open cohort study.

**Setting** Routinely collected data from 1457 general practices in England contributing to the QResearch database: 1094 were used to develop the scores and a separate set of 363 were used to validate the scores.

**Participants** 11.5 million people aged 25-84 and free of diabetes at baseline: 8.87 million in the derivation cohort and 2.63 million in the validation cohort.

**Methods** Cox proportional hazards models were used in the derivation cohort to derive separate risk equations in men and women for evaluation at 10 years. Risk factors considered included those already in QDiabetes (age, ethnicity, deprivation, body mass index, smoking, family history of diabetes in a first degree relative, cardiovascular disease, treated hypertension, and regular use of corticosteroids) and new risk factors: atypical antipsychotics, statins, schizophrenia or bipolar affective disorder, learning disability, gestational diabetes, and polycystic ovary syndrome. Additional models included fasting blood glucose and glycated haemoglobin (HBA1c). Measures of calibration and discrimination were determined in the validation cohort for men and women separately and for individual subgroups by age group, ethnicity, and baseline disease status.

**Main outcome measure** Incident type 2 diabetes recorded on the general practice record.

**Results** In the derivation cohort, 178 314 incident cases of type 2 diabetes were identified during follow-up arising from 42.72 million person years of observation. In the validation cohort, 62 326 incident cases of type 2 diabetes were identified from 14.32 million person years of observation. All new risk factors considered met our model inclusion criteria. Model A included age, ethnicity, deprivation, body mass index, smoking, family history of diabetes in a first degree relative, cardiovascular disease, treated hypertension, and regular use of corticosteroids, and new risk factors: atypical antipsychotics, statins, schizophrenia or bipolar affective disorder, learning disability, and gestational diabetes and polycystic ovary syndrome in women. Model B included the same variables as model A plus fasting blood glucose. Model C included HBA1c instead of fasting blood glucose. All three models had good calibration and high levels of explained variation and discrimination. In women, model B explained 63.3% of the variation in time to diagnosis of type 2 diabetes (R^2^), the D statistic was 2.69 and the Harrell’s C statistic value was 0.89. The corresponding values for men were 58.4%, 2.42, and 0.87. Model B also had the highest sensitivity compared with current recommended practice in the National Health Service based on bands of either fasting blood glucose or HBA1c. However, only 16% of patients had complete data for blood glucose measurements, smoking, and body mass index.

**Conclusions** Three updated QDiabetes risk models to quantify the absolute risk of type 2 diabetes were developed and validated: model A does not require a blood test and can be used to identify patients for fasting blood glucose (model B) or HBA1c (model C) testing. Model B had the best performance for predicting 10 year risk of type 2 diabetes to identify those who need interventions and more intensive follow-up, improving on current approaches. Additional external validation of models B and C in datasets with more completely collected data on blood glucose would be valuable before the models are used in clinical practice.

## Introduction

Diabetes risk assessment tools are used to identify people at increased risk of diabetes so they can have blood tests, and to target interventions to reduce risk. The first QDiabetes model to estimate 10 year risk of type 2 diabetes was published in 2009.^1^ Since then it has been updated regularly and recalibrated to the latest version of the QResearch database; the age range across which it applies has also been extended, from 25-79 to 25-84 years, smoking is assessed at five levels instead of two, and the Townsend score has been updated using most recent values from the 2011 census.^2^ ^3^ ^4^ This helps ensure that the algorithm reflects the changes in population characteristics (such as changes in prevalence of smoking, body mass index, and incidence of type 2 diabetes) and improvements in data quality such as improved recording of risk factors or ascertainment of diabetes. The QDiabetes algorithms have been validated by ourselves and others in independent groups of patients using UK primary care databases such as QResearch,^5^ Clinical Practice Research Datalink (CPRD),^5^ and The Health Improvement Network (THIN).^6^ The algorithms have been independently and externally validated in international populations and compared with other diabetes risk prediction models and been shown to have best performance.^7^ The use of QDiabetes has also been evaluated in observational studies and systematic reviews.^8^ ^9^ ^10^ ^11^

QDiabetes is now integrated into leading UK general practice computer systems and used within the UK National Health Service. It is recommended in the NHS Health Checks and National Institute for Health and Care Excellence guidance on the prevention of type 2 diabetes in people at high risk.^12^ ^13^ It is also used in occupational health settings and internationally through the publicly available QDiabetes website (www.qdiabetes.org). A recent update to the NICE guideline on diabetes prevention in 2017 has highlighted several conditions associated with increased diabetes risk that may not be fully captured by QDiabetes.^14^ These include polycystic ovary syndrome, gestational diabetes, learning disabilities, and mental health problems.^15^ Furthermore, there is now good evidence from both clinical trials and observational studies that atypical antipsychotics and statins are associated with an increased risk of diabetes.^16^ ^17^ ^18^ ^19^ ^20^ ^21^ ^22^ ^23^ These factors are not specifically identified within QDiabetes, which may result in under-estimation of risk in the relevant patient groups.

Once patients with an increased risk of developing diabetes have been identified using a diabetes risk assessment tool such as QDiabetes, then guidelines recommend they undergo a blood glucose test, either for glycated haemoglobin (HBA1c) or fasting blood glucose.^12^ ^13^ ^24^ This is to determine who already has diabetes; who is at high risk of progression to type 2 diabetes, and who is at moderate risk.^13^ International guidelines differ about which thresholds of fasting blood glucose and HBA1c to use to define the high risk group, mainly because of a lack of population based data on which to base the analyses.^25^ ^26^ For example, American guidelines recommend a fasting blood glucose concentration of 5.6-6.9 mmol/L or HBA1c value of 39-46 mmol/mol (5.7-6.4%). UK guidelines recommend a fasting blood glucose concentration of 5.5-6.9 mmol/L or HBA1c value of 42-47 mmol/mol (6.0-6.4%).^12^

Widely used risk assessment tools do not incorporate the results of either blood test, making it difficult to provide patients with an accurate estimation of their absolute level of risk after a blood test. We therefore derived and validated a new version of the equation (QDiabetes-2018) to determine whether these factors should be incorporated into the equation and how they could be used to improve the estimation of diabetes risk and communication with patients as well as improve the design of population based diabetes risk assessment strategies.

## Methods

### Study design and data source

We undertook a cohort study in a large population of primary care patients in England who were registered with practices contributing to the QResearch database (version 42). EMIS Health is the leading commercial supplier of general practice computer systems in the UK and is used by approximately 4400 practices. This is around 58% of all 7613 general practices in England (NHS Information Centre, March 2016). Of these, 1503 (34.2%) contribute to the QResearch database. We included all English practices contributing to QResearch who had been using their EMIS Health computer system for at least a year. We randomly allocated three quarters of practices to the derivation dataset and the remaining quarter to a validation dataset. We identified an open cohort of patients aged 25-84 years registered with practices between 1 January 2005 and 31 December 2016. We excluded patients who did not have a postcode related Townsend score (these usually result from patients moving to newly built houses with new postcodes not yet linked to deprivation data, or from patients being homeless or not having a permanent residence) and those with pre-existing type 1 or type 2 diabetes. We also excluded those with a fasting blood glucose concentration of 7 mmol/L or more or HBA1c value of 48 mmol/mol or more as these patients might be in the process of having further tests to confirm a diagnosis of diabetes. For each patient we determined an entry date to the cohort, which was the latest of the following: 25th birthday, date of registration with the practice plus one year, date on which the practice computer system was installed plus one year, and the beginning of the study period (1 January 2005). Patients were censored at the earliest date of the diagnosis of type 2 diabetes, death, deregistration with the practice, last upload of computerised data, or the study end date (31 December 2016).

### Outcomes

Our primary outcome measure was the first (incident) diagnosis of type 2 diabetes mellitus as recorded on the general practice computer records. We identified patients with diabetes by searching the electronic health record for diagnostic Read codes for diabetes (C10%). As in other studies, we classified patients as having type 2 diabetes if they had a diagnosis of diabetes and had not been prescribed insulin aged less than 35 years.^1^ ^27^ ^28^

### Predictor variables

We examined the following predictor variables based on established risk factors already included in the current version of QDiabetes-2017 and new candidate variables highlighted in the literature or NICE guidelines. Where diagnoses are mentioned, these relate to diagnostic codes recorded in the patients’ electronic health record on or before the study entry date.

#### Existing variables from QDiabetes (current 2017 version)

• Age at study entry (baseline)
• Ethnicity (nine categories)
• Deprivation (as measured by the Townsend score, where higher values indicate higher levels of material deprivation)
• Body mass index
• Smoking status: non-smoker, former smoker, light smoker (1-9/day), moderate smoker (10-19/day), heavy smoker (≥20/day)
• Family history of diabetes in a first degree relative
• Cardiovascular disease (ischaemic heart disease, stroke, or transient ischaemic attack)
• Treated hypertension (diagnosis of hypertension and current treatment with at least one antihypertensive drug)
• Corticosteroids (*British National Formulary* chapter 6.3.2, including oral or injections of systemic prednisolone, betamethasone, cortisone, depo-medrone, dexamethasone, deflazacort, efcortesol, hydrocortisone, methylprednisolone, or triamcinolone)

#### New or amended risk factors considered

• Diagnosis of schizophrenia or bipolar affective disorder
• Learning disabilities
• Diagnosis of gestational diabetes
• Diagnosis of polycystic ovary syndrome
• Prescribed second generation “atypical” antipsychotics (including amisulpride, aripiprazole, clozapine, lurasidone, olanzapine, paliperidone, quetiapine, risperidone, sertindole, and zotepine)
• Prescribed statins
• Fasting blood glucose level
• Glycated haemoglobin (HBA1c) value

From the general practice record we extracted data on the demographic factors, clinical diagnoses, and clinical values. We selected the closest value to cohort entry for body mass index, smoking status, fasting blood glucose level, and HBA1c level, restricting to values recorded before baseline. Use of drugs at baseline was defined as at least two prescriptions in total, with the most recent one no more than 28 days before the date of entry to the cohort. All other predictor variables were based on the latest information recorded in the general practice record before entry to the cohort.

### Derivation and validation of the models

We developed and validated the risk prediction equations using established methods.^10^ ^29^ ^30^ ^31^ ^32^ An initial analysis was performed based on patients with complete data. We used multiple imputation with chained equations to replace missing values for body mass index, HBA1c, fasting blood glucose, and smoking status and used these values in our main analyses.^33^ ^34^ ^35^ So that the imputed values would better match the distribution of observed values, we log transformed values for continuous variables that were not normally distributed for inclusion in the imputation model. In that model we included all predictor variables, along with the Nelson-Aalen estimator of the baseline cumulative hazard, and the outcome indicator. We carried out five imputations, as this has a relatively high efficiency^36^ and was a pragmatic approach accounting for the size of the datasets and capacity of the available servers and software. The same imputed dataset was used for all the derivation analyses.

To estimate the coefficients for each risk factor in men and women separately we used Cox’s proportional hazards models. We used Rubin’s rules to combine the results across the imputed datasets.^37^ We used fractional polynomials^38^ to model non-linear risk relations with continuous variables. We selected second degree fractional polynomial terms derived using the data from patients with recorded values.^39^ Before running the Cox models we applied the fractional polynomial terms to the imputed data. Initially we fitted full models. For consistency, we included variables from existing QDiabetes models and then retained additional variables if they had an adjusted hazard ratio of less than 0.90 or more than 1.10 (for binary variables) and were statistically significant at the 0.01 level. We used these criteria in conjunction with clinical judgment to ensure that candidate variables were likely to be clinically important and to reduce the possibility of including weak or uninformative predictors that could lead to model over-fitting and optimism.^40^ We examined interactions between new predictor variables and age at study entry and included significant interactions in the final models along with interactions already included in the current version of QDiabetes. To compare fit and performance of different models in the derivation cohort we used Akaikes Information Criterion.

We developed three main models. Model A included the variables in the existing QDiabetes models and the additional variables that met our inclusion criteria but did not include either fasting blood glucose or HBA1c. Model B is the same as model A except that it included fasting blood glucose but not HBA1c. Model C is the same as model A except that it included HBA1c but not fasting blood glucose.

We used the regression coefficients for each variable from the final models as weights, which we combined with non-parametric estimates of the baseline survivor function,^41^ evaluated for each year up to 10 years to derive risk equations over 10 years of follow-up.^42^ This enabled us to derive risk estimates for each year of follow-up, with a specific focus on 10 year risk estimates. We estimated the baseline survivor function based on zero values of centred continuous variables, with all binary predictor values set to zero.

### Validation of the models

In the validation cohort we used multiple imputation to replace missing values for body mass index, fasting blood glucose, HBA1c, and smoking status. We carried out five imputations. The risk equations for men and women obtained from the derivation cohort were then applied to the validation cohort and measures of discrimination calculated. As in previous studies,^5^ we calculated the D statistic^43^ (a measure of discrimination where higher values indicate better discrimination), R^2^ value (explained variation where higher values indicate a greater proportion of variation explained by the model in time to diagnosis of type 2 diabetes^44^) based on Royston’s D statistic, and Harrell’s C statistic at 10 years and combined these across datasets using Rubin’s rules. Harrell’s C statistic^45^ is a measure of discrimination similar to the receiver operating characteristic statistic but takes account of the censored nature of the data.

Calibration was assessed by comparing the mean predicted risks at 10 years with the observed risk by 10th of predicted risk. The observed risks were obtained using the Kaplan-Meier estimates evaluated at 10 years. We also evaluated performance by subgroups for each age band (<40, 40-59, ≥60 years), ethnic minority group, and comorbidity and treatment group. We calculated calibration slopes. Performance was also evaluated by calculating Harrell’s C statistics in individual general practices and combining the results using meta-analytical techniques.^46^

By applying each equation to the validation dataset we compared performance statistics for the new QDiabetes-2018 models with the latest version of QDiabetes (2017 version).

### Risk stratification

For model A we calculated sensitivity, specificity, and observed risks at different risk thresholds in the validation cohort.

We also compared performance of the models with current recommendations from the “two step” approach recommended in the NICE guidance “Preventing type 2 diabetes risk”^13^ and the NHS Health Checks best practice guideline.^12^ Step 1 currently involves using a risk assessment tool such as QDiabetes to identify “high risk” patients, where high risk is defined for QDiabetes as those who have a 10 year risk of type 2 diabetes of 5.6% or greater.^12^ This threshold appears to have been selected predominantly to optimise sensitivity (ie, to avoid missing cases of type 2 diabetes). Step 2 involves a blood test for those identified at high risk to assess whether they have undiagnosed type 2 diabetes, and in the remaining patients to more accurately stratify their risk of progression to diabetes. This blood test can be either for fasting blood glucose or for HBA1c in high risk patients to classify patients into one of three groups: fasting blood glucose ≥7 mmol/L or HBA1c ≥48 mmol/mol = diagnosis of diabetes (or further testing required for confirmation if patient has no symptoms); fasting blood glucose 5.5-6.9 mmol/L or HBA1c 42-47 mmol/mol = “high risk of diabetes” for intensive lifestyle advice or intervention programme; and fasting blood glucose <5.5 mmol/L or HBA1c <42 mmol/mol = “moderate risk of diabetes” for simple lifestyle advice.

The updated QDiabetes models were designed to support such an approach, with model A intended to identify patients with an increased risk for whom a blood test could be done. The two further models (model B including fasting blood glucose and model C including HBA1c) could then be used to refine the risk assessment tool once the relevant blood test result was available. Risk assessment at this point could also allow communication of a more accurate risk estimate for patients to inform their decision making and management plans.

To compare performance of the models with current recommendations, we calculated the sensitivity for four different strategies for classifying patients as high risk of progression for diabetes using the validation cohort. Patients were classified as at high risk if they had an initial 10 year QDiabetes risk score of 5.6% or more (using model A) and (i) they had a fasting blood glucose concentration between 5.5 and 6.9 mmol/L (strategy 1), (ii) an HBA1c value between 42 and 47 mmol/mol (strategy 2), (iii) a risk score in the top 28% of risk scores using model B (which includes fasting blood glucose values) to correspond to the number of high risk patients for strategy 1 (strategy 3), and (iv) a risk score in the top 28% of risk scores using model C (which includes HBA1c values) to correspond to the number of high risk patients for strategies 1 and 3 (strategy 4).

### Decision curve analysis

To evaluate the net benefits of the updated risk equations we used decision curve analysis in the validation cohort.^47^ ^48^ ^49^ This approach assesses the benefits of correctly detecting people who will develop type 2 diabetes compared with the harms from a false positive classification (which could lead to unnecessary intervention). The net benefit of a risk equation at a given risk threshold is given by calculating the *difference between the proportion of true positives* and the *proportion of false positives multiplied by the odds defined by the risk threshold value*.^48^ We calculated the net benefits of models A, B, and C across a range of threshold probabilities and compared these with alternative strategies, such as assuming no patients will develop type 2 diabetes (no intervention) or assuming all patients will develop type 2 diabetes (intervention in all patients). In general, the strategy with the highest net benefit at any given risk threshold is considered to have the most clinical value.

To maximise the power and generalisability of the results, we used all the relevant patients on the database. Stata (version 14) was used for all analyses. We adhered to the TRIPOD statement for reporting.^40^

### Patient involvement

Since the original publication of QDiabetes-2009 there has been public stakeholder discussion about methods for assessment of diabetes risk as part of the development of the NICE guidance and NHS Health Checks.^50^ We therefore decided to focus on issues already identified in NICE guidance and the literature. We decided it would be more effective to discuss the addition of new variables once the paper was published and the relative importance of individual risk factors has been quantified. Given the widespread implementation of QDiabetes within the NHS and its inclusion in guidelines, this would allow for feedback from stakeholders (including patient groups and charities) as to which changes would be most beneficial and how improvements might be implemented.

## Results

### Study population

Overall, 1457 QResearch practices in England met our inclusion criteria (96.9% of all practices contributing to QResearch). Of these, three quarters (n=1094) were randomly assigned to the derivation dataset, with the remaining quarter (n=363) assigned to a validation cohort. We identified 8 640 363 patients in the derivation cohort aged 25-84 years of whom we sequentially excluded 26 602 (0.3%) who did not have a recorded Townsend score, 34 195 (0.4%) who had a diagnosis of type 1 diabetes at baseline, 342 858 (4.0%) who had a diagnosis of type 2 diabetes at baseline, 23 522 (0.3%) with a fasting blood glucose concentration of 7 mmol/L or more at baseline, and 26 481 (0.3%) with a HBA1c value of 48 mmol/mol or more at baseline. This left 8 186 705 for the derivation analysis.

We identified 2 779 075 patients in the validation cohort aged 25-84 years of whom we sequentially excluded 7971 (0.3%) who did not have a recorded Townsend score, 11 076 (0.4%) who had a diagnosis of type 1 diabetes at baseline, 113 653 (4.1%) who had a diagnosis of type 2 diabetes at baseline, 7758 (0.3%) with a fasting blood glucose of 7 mmol/L or more, and 8677 (0.3%) with a HBA1c concentration of 48 mmol/mol or more at baseline. This left 2 629 940 for the validation analysis.

### Baseline characteristics

Table 1 shows the baseline characteristics of men and women in the derivation and validation cohorts. In the derivation cohort, the mean age was 44.9 (SD 15.3) years, and 4 062 142 (49.6%) were men. Self assigned ethnicity was recorded in 5 933 548 (72.5%), smoking status in 7 834 644 (95.7%), body mass index in 6 482 691 (79.2%), fasting blood glucose in 1 189 398 (14.5%), and HBA1c in 506 776 (6.2%). In total, 6 453 196 (78.8%) had complete information for smoking status and body mass index, and 1 367 483 (16.7%) had complete information for smoking, body mass index, and either fasting blood glucose or HBA1c. These values were similar to corresponding values in the validation cohort (table 1).

**Table 1 tbl1:** Baseline characteristics of adults aged 25-84 without diabetes at study entry. Values are numbers (percentages) of patients unless stated otherwise

Characteristics	Derivation cohort (n=8 186 705)	Validation cohort (n=2 629 940)
Men	4 062 142 (49.6)	1 307 505 (49.7)
Women	4 124 563 (50.4)	1 322 435 (50.3)
Mean (SD) age (years)	44.9 (15.3)	45.6 (15.5)
Mean (SD) Townsend score	0.5 (3.3)	0.2 (3.2)
Body mass index recorded	6 482 691 (79.2)	2 03 3369 (77.3)
Mean (SD) body mass index	26.0 (5.0)	26.0 (5.0)
Fasting blood glucose recorded	1 189 398 (14.5)	373 808 (14.2)
Mean (SD) fasting blood glucose (mmol/L)	5.0 (0.6)	5.0 (0.6)
HBA1c recorded	506 776 (6.2)	161 966 (6.2)
Mean (SD) HBA1c (mmol/mol)	37.2 (4.5)	37.3 (4.4)
Complete data*	6 453 196 (78.8)	2 024 909 (77.0)
Complete data†	1 367 483 (16.7)	416 142 (15.8)
Ethnic origin:		
Ethnicity recorded	5 933 548 (72.5)	1 870 332 (71.1)
White or not recorded	7 136 377 (87.2)	2 323 760 (88.4)
Indian	188 049 (2.3)	58 084 (2.2)
Pakistani	101 231 (1.2)	33 954 (1.3)
Bangladeshi	81 834 (1.0)	22 148 (0.8)
Other Asian	122 981 (1.5)	38 222 (1.5)
Caribbean	80 657 (1.0)	22 379 (0.9)
Black African	179 423 (2.2)	48 446 (1.8)
Chinese	65 999 (0.8)	15 947 (0.6)
Other	230 154 (2.8)	67 000 (2.5)
Smoking status:		
Smoking recorded	7 834 644 (95.7)	2 520 127 (95.8)
Non-smoker	4 441 795 (54.3)	1 422 825 (54.1)
Former smoker	1 518 799 (18.6)	502 297 (19.1)
Light smoker	1 098 645 (13.4)	344 874 (13.1)
Moderate smoker	485 756 (5.9)	155 933 (5.9)
Heavy smoker	289 649 (3.5)	94 198 (3.6)
Medical characteristics:		
Family history of diabetes	1 21 8682 (14.9)	379 889 (14.4)
Treated hypertension	737 303 (9.0)	249 614 (9.5)
Cardiovascular disease	290 345 (3.5)	101 370 (3.9)
Schizophrenia or bipolar affective disorder	62 014 (0.8)	19 619 (0.7)
Learning disability	56 092 (0.7)	18 458 (0.7)
Gestational diabetes‡	17 214 (0.4)	5201 (0.2)
Polycystic ovary syndrome‡	81 164 (2.0)	24 217 (0.9)
Current drugs:		
Statins	526 969 (6.4)	173 528 (6.6)
Atypical antipsychotics	58 655 (0.7)	19 776 (0.8)
Corticosteroids	238 683 (2.9)	83 760 (3.2)

*Complete data for body mass index and smoking.

†Complete data for body mass index, smoking, and either HBA1c or fasting glucose.

‡% of all women.

Table 1 also shows medical characteristics at study entry. For the new variables of interest: 58 655 (0.7%) patients in the derivation cohort were prescribed atypical antipsychotics, 526 969 (6.4%) were prescribed statins, schizophrenia or bipolar affective disorder was recorded in 62 014 (0.8%), learning disability was recorded in 56 092 (0.7%), gestational diabetes in 17 214 (0.4% of women), and polycystic ovary syndrome in 81 164 (2.0% of women).

Supplementary table 1a shows the distribution of risk factors by ethnic group in the derivation cohort. Testing for fasting blood glucose and HBA1c was higher among all non-white ethnic groups other than Chinese compared with the white or not recorded group. Compared with the other ethnic groups, people of South Asian and Caribbean origin tended to have marginally higher mean HBA1c values, and higher proportions had a family history of diabetes.

Supplementary table 1b shows similar information for patients with fasting blood glucose or HBA1c recorded compared with those without a value for either test.

### Incidence of type 2 diabetes

Table 2 shows the numbers of patients with a new diagnosis of type 2 diabetes during follow-up in the derivation and validation cohorts. In the derivation cohort, we identified 178 314 incident cases of type 2 diabetes arising from 42.7 million person years of observation. Supplementary table 2 shows a breakdown by nine ethnic groups. For example, 6181 incident cases of type 2 diabetes for men and women of Indian ethnicity arose from 795 000 person years of observation.

**Table 2 tbl2:** Incidence of type 2 diabetes per 1000 person years with 95% confidence intervals in derivation and validation cohorts

Variables	Derivation cohort				Validation cohort		
	Incident cases	Person years (000s)	Incidence per 1000 (95% CI)		Incident cases	Person years (000s)	Incidence per 1000 (95% CI)
Total	178 314	42 718	4.17 (4.15 to 4.19)		62 326	14 317	4.35 (4.32 to 4.39)
Women	77 895	21 561	3.61 (3.59 to 3.64)		27 311	7242	3.77 (3.73 to 3.82)
Men	100 419	21 157	4.75 (4.72 to 4.78)		35 015	7076	4.95 (4.90 to 5.00)
Age band (years):							
25-29	2351	5042	0.47 (0.45 to 0.49)		809	1593	0.51 (0.47 to 0.54)
30-34	6058	5442	1.11 (1.09 to 1.14)		2000	1699	1.18 (1.13 to 1.23)
35-39	11 419	5580	2.05 (2.01 to 2.08)		3952	1807	2.19 (2.12 to 2.26)
40-44	16 770	5336	3.14 (3.10 to 3.19)		5519	1778	3.10 (3.02 to 3.19)
45-49	20 199	4502	4.49 (4.42 to 4.55)		6863	1509	4.55 (4.44 to 4.66)
50-54	21 698	3776	5.75 (5.67 to 5.82)		7203	1289	5.59 (5.46 to 5.72)
55-59	24 688	3668	6.73 (6.65 to 6.82)		8862	1305	6.79 (6.65 to 6.93)
60-64	22 518	2906	7.75 (7.65 to 7.85)		8021	1031	7.78 (7.61 to 7.95)
65-69	20 039	2315	8.66 (8.54 to 8.78)		7256	827	8.78 (8.58 to 8.98)
70-74	16 102	1829	8.81 (8.67 to 8.94)		5844	655	8.92 (8.69 to 9.15)
75-79	10 818	1399	7.73 (7.59 to 7.88)		3892	493	7.89 (7.65 to 8.14)
80-84	5654	923	6.13 (5.97 to 6.29)		2105	333	6.33 (6.07 to 6.61)
Medical characteristics:							
Family history of diabetes	44 822	6360	7.05 (6.98 to 7.11)		15 951	2104	7.58 (7.46 to 7.70)
Treated hypertension	54 367	4491	12.11 (12.00 to 12.21)		19 202	1574	12.20 (12.03 to 12.38)
Cardiovascular disease	20 206	1583	12.76 (12.59 to 12.94)		7310	570	12.82 (12.53 to 13.11)
Schizophrenia or bipolar affective disorder	2506	272	9.21 (8.86 to 9.58)		856	89	9.61 (8.99 to 10.28)
Learning disability	1313	251	5.22 (4.95 to 5.51)		426	84	5.05 (4.59 to 5.56)
Gestational diabetes	1123	63	17.75 (16.74 to 18.82)		345	19	17.72 (15.95 to 19.69)
Polycystic ovary syndrome	1243	322	3.85 (3.65 to 4.08)		428	99	4.34 (3.94 to 4.77)
Current drugs:							
Statins	37 231	2,879	12.93 (12.80 to 13.06)		12 603	977	12.89 (12.67 to 13.12)
Atypical antipsychotics	2350	247	9.50 (9.12 to 9.89)		832	84	9.86 (9.22 to 10.56)
Corticosteroids	13 390	1579	8.48 (8.34 to 8.63)		4849	574	8.45 (8.21 to 8.69)

The median follow-up in the derivation cohort was 3.90 years (interquartile range 1.54 to 8.50). Overall, 2 027 279 patients had 10 or more years of follow-up. The median follow-up in the validation cohort was 4.22 years (1.57 to 9.25). Overall, 602 661 patients had 10 or more years of follow-up.

### Predictor variables

Table 3 shows the adjusted hazard ratios for models A, B, and C in women in the derivation cohort. Table 4 shows the corresponding values for men.

**Table 3 tbl3:** Adjusted hazard ratios (95% confidence interval) for type 2 diabetes in women in the derivation cohort for models A, B, and C

Variables	Adjusted hazard ratio (95% CI)		
	Model A	Model B	Model C
Townsend score (5 unit increase)	1.21 (1.19 to 1.22)	1.20 (1.18 to 1.21)	1.20 (1.18 to 1.21)
Ethnic group:			
White or not recorded	1.00	1.00	1.00
Indian	2.91 (2.80 to 3.03)	2.69 (2.57 to 2.81)	1.82 (1.74 to 1.91)
Pakistani	3.83 (3.66 to 4.01)	3.49 (3.29 to 3.71)	2.19 (2.05 to 2.33)
Bangladeshi	6.07 (5.77 to 6.38)	4.45 (4.20 to 4.73)	3.30 (3.10 to 3.52)
Other Asian	3.09 (2.92 to 3.26)	2.63 (2.48 to 2.79)	2.04 (1.92 to 2.17)
Caribbean	1.52 (1.45 to 1.60)	1.62 (1.54 to 1.71)	1.13 (1.07 to 1.18)
Black African	1.33 (1.26 to 1.40)	1.61 (1.53 to 1.70)	1.01 (0.96 to 1.07)
Chinese	2.41 (2.15 to 2.72)	2.12 (1.88 to 2.39)	1.77 (1.53 to 2.05)
Other	1.44 (1.37 to 1.52)	1.50 (1.42 to 1.58)	1.19 (1.12 to 1.25)
Smoking status:			
Non-smoker	1.00	1.00	1.00
Former smoker	1.07 (1.05 to 1.09)	1.04 (1.01 to 1.06)	1.07 (1.05 to 1.09)
Light smoker	1.33 (1.30 to 1.36)	1.25 (1.22 to 1.29)	1.16 (1.13 to 1.19)
Moderate smoker	1.43 (1.38 to 1.48)	1.36 (1.32 to 1.41)	1.16 (1.12 to 1.21)
Heavy smoker	1.71 (1.65 to 1.77)	1.55 (1.48 to 1.62)	1.36 (1.29 to 1.43)
Medical characteristics*:			
Family history of diabetes†	1.70 (1.67 to 1.74)	1.57 (1.53 to 1.61)	1.56 (1.52 to 1.59)
Treated hypertension	1.55 (1.53 to 1.58)	1.33 (1.30 to 1.35)	1.50 (1.47 to 1.53)
Cardiovascular disease	1.19 (1.16 to 1.23)	1.22 (1.18 to 1.26)	1.18 (1.14 to 1.22)
Schizophrenia or bipolar affective disorder	1.30 (1.21 to 1.39)	1.18 (1.08 to 1.28)	1.37 (1.27 to 1.49)
Learning disability†	1.32 (1.19 to 1.46)	1.57 (1.41 to 1.76)	1.34 (1.20 to 1.48)
Gestational diabetes	4.59 (4.32 to 4.88)	2.91 (2.71 to 3.13)	3.08 (2.87 to 3.31)
Polycystic ovary syndrome	1.41 (1.33 to 1.49)	1.43 (1.35 to 1.51)	1.40 (1.31 to 1.50)
Current drugs*:			
Statins†	1.93 (1.84 to 2.03)	1.79 (1.70 to 1.88)	1.58 (1.49 to 1.68)
Atypical antipsychotics†	1.74 (1.60 to 1.89)	1.61 (1.46 to 1.76)	1.73 (1.56 to 1.92)
Corticosteroids	1.31 (1.28 to 1.34)	1.46 (1.42 to 1.50)	1.18 (1.15 to 1.21)

For fractional polynomial terms and age interactions see figures as well as footnotes.

*Compared with patients without this characteristic.

†Interaction with age; hazard ratio evaluated at mean age.

Model A also includes fractional polynomial terms for age (age^0.5^, age^3^) and body mass index (BMI, BMI^3^) with interaction terms between age and atypical antipsychotics, statins, learning disability, body mass index, and family history of diabetes.

Model B same as model A plus fractional polynomial terms for fasting blood glucose (fasting glucose^−1^, fasting glucose^−1^ log (fasting glucose)) and interaction terms between age and fasting glucose.

Model C same as model A plus fractional polynomial terms for HBA1c (HBA1c^0.5^, HBA1c) and interaction terms between age and HBA1c.

**Table 4 tbl4:** Adjusted hazard ratios (95% confidence interval) for type 2 diabetes in men in the derivation cohort for models A, B, and C

Variables	Adjusted hazard ratio (95% CI)		
	Model A	Model B	Model C
Townsend score (5 unit increase)	1.14 (1.13 to 1.15)	1.14 (1.12 to 1.15)	1.13 (1.12 to 1.15)
Ethnic group:			
White or not recorded	1.00	1.00	1.00
Indian	3.00 (2.90 to 3.11)	2.74 (2.64 to 2.84)	1.97 (1.89 to 2.04)
Pakistani	3.63 (3.48 to 3.79)	3.80 (3.64 to 3.98)	2.30 (2.20 to 2.40)
Bangladeshi	5.33 (5.09 to 5.59)	4.40 (4.14 to 4.68)	2.99 (2.82 to 3.18)
Other Asian	3.13 (2.98 to 3.28)	2.82 (2.67 to 2.99)	2.16 (2.04 to 2.28)
Caribbean	1.60 (1.52 to 1.68)	1.68 (1.59 to 1.78)	1.23 (1.15 to 1.32)
Black African	2.01 (1.92 to 2.11)	2.36 (2.23 to 2.49)	1.46 (1.39 to 1.54)
Chinese	1.99 (1.78 to 2.23)	1.90 (1.67 to 2.16)	1.41 (1.22 to 1.62)
Other	1.52 (1.45 to 1.59)	1.62 (1.55 to 1.70)	1.25 (1.18 to 1.31)
Smoking status:			
Non-smoker	1.00	1.00	1.00
Former smoker	1.18 (1.16 to 1.20)	1.12 (1.10 to 1.14)	1.12 (1.10 to 1.14)
Light smoker	1.38 (1.35 to 1.40)	1.36 (1.34 to 1.39)	1.16 (1.13 to 1.18)
Moderate smoker	1.38 (1.34 to 1.42)	1.39 (1.35 to 1.44)	1.11 (1.07 to 1.16)
Heavy smoker	1.57 (1.52 to 1.62)	1.53 (1.48 to 1.58)	1.22 (1.18 to 1.26)
Medical characteristics*:			
Family history of diabetes†	1.91 (1.88 to 1.95)	1.78 (1.74 to 1.81)	1.76 (1.72 to 1.80)
Treated hypertension	1.40 (1.37 to 1.42)	1.28 (1.26 to 1.30)	1.39 (1.36 to 1.43)
Cardiovascular disease	1.22 (1.20 to 1.25)	1.24 (1.21 to 1.27)	1.16 (1.13 to 1.19)
Schizophrenia or bipolar affective disorder	1.26 (1.18 to 1.34)	1.24 (1.16 to 1.34)	1.33 (1.24 to 1.43)
Learning disability†	1.26 (1.16 to 1.38)	1.49 (1.37 to 1.63)	1.30 (1.17 to 1.43)
Current drugs‡:			
Statins†	1.79 (1.74 to 1.86)	1.67 (1.61 to 1.74)	1.53 (1.48 to 1.58)
Atypical antipsychotics†	1.52 (1.40 to 1.65)	1.56 (1.43 to 1.70)	1.58 (1.44 to 1.72)
Corticosteroids	1.25 (1.21 to 1.28)	1.41 (1.36 to 1.45)	1.15 (1.11 to 1.18)

For fractional polynomial terms and age interactions see figures as well as footnotes.

*Compared with patients without this characteristic.

†Interaction with age; hazard ratio evaluated at mean age.

Model A also includes fractional polynomial terms for age (ln(age), age^3^) and body mass index (BMI,^2^ BMI^3^) and interactions between age and atypical antipsychotics, statins, learning disability, body mass index, family history of diabetes.

Model B is same as model A plus fractional polynomial terms for fasting blood glucose (fasting glucose^−0.5^, fasting glucose^−0.5^ log (fasting glucose)) and interactions between age and fasting glucose.

Model C is same as model A plus fractional polynomial terms for HBA1c (HBA1c^0.5^, HBA1c) and interactions between age and HBA1c.

Of the new risk factors, all met our model inclusion criteria. Model A includes the variables: age, ethnicity, deprivation, body mass index, smoking status, family history of diabetes in a first degree relative, cardiovascular disease, treated hypertension, corticosteroids, atypical antipsychotics, statins, schizophrenia or bipolar affective disorder, and learning disability. The model in women also included gestational diabetes and polycystic ovary syndrome.

Model B is the same as model A except it includes fasting blood glucose. Model C is the same as model A except it includes HBA1c.

Supplementary figure S1 shows graphs of the adjusted hazard ratios for models A and B for the fractional polynomial terms for age and body mass index as well as the interaction terms between age and relevant predictor variables as listed in the footnotes of tables 3 and 4[Table tbl3 tbl4]. Supplementary figure S1 also shows graphs of the adjusted hazard ratios for fasting blood glucose in model B and HBA1c in model C and their interactions with age.

For the new variables of interest in model A, atypical antipsychotics were associated with a 74% (95% confidence interval 60% to 89%) increased risk of type 2 diabetes in women and a 52% (40% to 65%) increased risk for men; statins were associated with a 93% (84% to 103%) increased risk in women and a 79% (74% to 86%) increased risk in men; schizophrenia or bipolar affective disorder was associated with a 30% (21% to 39%) increased risk in women and a 26% (18% to 34%) increased risk in men; learning disability was associated with a 32% (19% to 46%) increased risk in women and a 26% (16% to 38%) increased risk in men. Gestational diabetes was associated with a 359% (332% to 388%) increased risk in women and polycystic ovary syndrome was associated with a 41% (33% to 49%) increased risk. Where there were age interactions, these values relate to risks evaluated at the mean ages.

The footnotes for tables 3 and 4[Table tbl3 tbl4] contain the full list of age interactions for each model. In both men and women, among the new variables there were statistically significant interactions between age and learning disability, age and atypical antipsychotics, age and statins, and age and fasting blood glucose (model B), and between age and HBA1c (model C). Hazard ratios for learning disability, atypical antipsychotics and statins, body mass index, and family history of diabetes were higher at younger ages compared with older ages (supplementary figure S1c-g). For example, for model B in men, statins were associated with a 141% increased risk at age 35, a 66% increased risk at age 45, a 30% increased risk at age 55, and a 15% increased risk at age 65 (supplementary figure S1d).

Overall the hazard ratios for models B and C tended to be lower than those for model A. The hazard ratios for non-white ethnic groups tended to be lower for model C than for model B. For example, the hazard ratio for Bangladeshi women was 6.07 (5.77 to 6.38) for model A, 4.45 (4.20 to 4.73) for model B, and 3.30 (3.10 to 3.52) for model C.

Supplementary tables 3 and 4 show the results of the complete case analysis for each of the three models. The adjusted hazard ratios for models A and B are broadly similar to the analysis based on imputed data.

### Validation

#### Discrimination

Table 5 shows the performance of each equation in the validation cohort for women and men for each of models A, B, and C compared with the current QDiabetes model. All models had good calibration and high levels of explained variation and discrimination. Model B had the best overall performance, followed by model C. Model A has a similar performance to the current QDiabetes models. Performance of all models was marginally better among women than among men.

**Table 5 tbl5:** Performance of models A, B, and C compared with current QDiabetes-2017 model in validation cohort in men and women

Statistics	Current QDiabetes-2017 model	Model A: QDiabetes-2018	Model B: QDiabetes-2018 (including FBG)	Model C: QDiabetes-2018 (including HBA1c)
Women:				
D statistic*	2.02 (2.00 to 2.04)	2.07 (2.05 to 2.09)	2.69 (2.65 to 2.73)	2.52 (2.47 to 2.57)
Harrell’s C*	0.831 (0.828 to 0.833)	0.834 (0.832 to 0.837)	0.889 (0.887 to 0.891)	0.878 (0.875 to 0.881)
R^2^ (%)†	49.3 (48.8 to 49.8)	50.5 (50.0 to 51.0)	63.3 (62.7 to 64.0)	60.3 (59.4 to 61.2)
Men:				
D statistic*	1.89 (1.88 to 1.91)	1.91 (1.89 to 1.93)	2.42 (2.40 to 2.45)	2.28 (2.20 to 2.36)
Harrell’s C*	0.813 (0.810 to 0.815)	0.814 (0.812 to 0.816)	0.866 (0.863 to 0.868)	0.855 (0.849 to 0.861)
R^2^ (%)†	46.1 (45.7 to 46.6)	46.6 (46.1 to 47.1)	58.4 (57.9 to 58.8)	55.5 (53.7 to 57.2)

FBG=fasting blood glucose; HBA1c=glycated haemoglobin.

*Measure of discrimination—higher values indicate better discrimination.

†Measures explained variation in time to diagnosis of diabetes —higher values indicate more explained variation.

In women, model A explained 50.5% of the variation in time to diagnosis of type 2 diabetes (R^2^), the D statistic was 2.07, and the Harrell’s C statistic was 0.834. The corresponding values for model A in men were 46.6%, 1.91, and 0.814.

In women, model B explained 63.3% of the variation in time to diagnosis of type 2 diabetes (R^2^), the D statistic was 2.69, and the Harrell’s C statistic was 0.889. The corresponding values for model B in men were 58.4%, 2.42, and 0.866.

In women, model C explained 60.3% of the variation in time to diagnosis of type 2 diabetes (R^2^), the D statistic was 2.52, and the Harrell’s C statistic was 0.878. The corresponding values for model C in men were 55.5%, 2.28, and 0.855.

In addition, we calculated Harrell’s C statistics for model B on the subgroup of patients with complete data for fasting blood glucose and for model C on those with complete data for HBA1c. The results for model B were 0.836 for women and 0.812 for men. The corresponding results for model C were 0.772 and 0.738.

Supplementary table 5 shows the D, R^2^, and Harrell’s C statistics for models A and B for women in various subgroups, including three age groups, ethnic groups, and those with specific morbidities. Supplementary table 6 shows the corresponding values for men.

The best performance by ethnic group was for model B among Chinese women (R^2^=68.0%, D=2.99, Harrell’s C=0.912). The poorest performance by ethnic group was for model A among Bangladeshi women (R^2^=35.6%, D=1.52, Harrell’s C=0.776). Performance values were highest in the youngest age group (<40 years) and lowest in the oldest age group (≥60 years) for both models.

Supplementary figure S2a-d shows plots of Harrell’s C statistic for models A and B in men and women across the 363 practices in the validation cohort. The plots show Harrell’s C values for each general practice versus the number of patients with a diagnosis of type 2 diabetes in each practice. Practices with fewer patients with a diagnosis of type 2 diabetes had wider variation in C statistic than practices with more diagnoses. For example, supplementary figure 2a shows the summary (average) C statistic for model A in women was 0.834 from a random effects meta-analysis. The I^2^ value (ie, the percentage of total variation in C statistic due to heterogeneity between practices) was 90.1%. The approximate 95% prediction interval for the true C statistic in women in a new practice was 0.72 to 0.94. Supplementary figure 2c shows the corresponding results for model B in women (summary C statistic=0.891, I^2^=77.5%, 95% prediction interval 0.83 to 0.96).

#### Calibration

In women, the mean 10 year predicted risk was 3.62% for model A and 3.42% for model B. The observed 10 year risk was 4.21% (95% confidence interval 4.16% to 4.26%). In men, the mean 10 year predicted risk was 4.97% for model A and 4.71% for model B. The observed 10 year risk was 5.56% (5.48% to 5.61%).

Figure 1 shows the mean predicted risks and observed risks at 10 years by 10th of predicted risk, applying models A, B, and C to all men and women in the validation cohort. Supplementary table 7 shows values of the calibration slope overall and by subgroup for models A and B. For example, the calibration slope for model A was 0.997 (0.986 to 1.008) in women and 0.986 (0.976 to 0.996) in men. For model B, the corresponding values were 0.993 (0.978 to 1.007) and 0.985 (0.975 to 0.996). The close correspondence between the mean predicted risks and the observed risks within each model 10th for each model indicates that the equations were well calibrated overall and by age group. Calibration within subgroups was variable, although it tended to be better for model B than for model A (see supplementary table 7).

**Figure f1:**
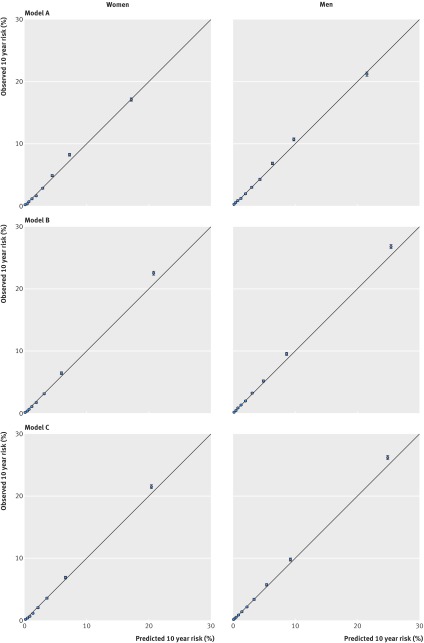
**Fig 1** Predicted and observed 10 year risk of diabetes using models A, B, and C

### Clinical use of QDiabetes

Figure 2 compares four strategies for identifying high risk patients based on current recommendations from the NHS Health Checks best practice guide (strategies 1 and 2) and risk assessment using models B and C in combination with model A (strategies 3 and 4). It shows that in the validation cohort for the patients identified as high risk using model A then strategy 3 (based on model B) was the most sensitive when equal sized groups are compared since it identified 28 953 (67.3%) of the 43 010 patients with a diagnosis of type 2 diabetes during 10 years follow-up who were classified as high risk at step 1 (and 49.8% of all 58 130 patients with a diagnosis of type 2 diabetes during 10 years follow-up in the whole validation cohort). Strategy 1 (based on a fasting blood glucose concentration of 5.5-6.9 mmol/L) identified 27 459 (63.8% of 43 010 and 47.2% of 58 130) and strategy 4 (based on model C) identified 27 061 (62.9% of 43 010 and 46.6% of 58 130) patients with a diagnosis of type 2 diabetes during 10 years follow-up. Strategy 2 (based on HBA1c values of 42-47 mmol/mol) identified a lower proportion of high risk patients (19.1%) and the least proportion of patients with a diagnosis of type 2 diabetes during 10 years follow-up, with only 20 037 (46.6% of 43 010 and 34.5% of 58 130) identified.

**Figure f2:**
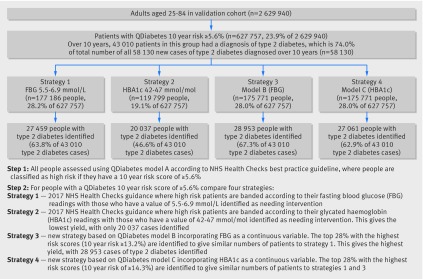
**Fig 2** Comparison of four strategies for identifying patients at high risk of developing diabetes. FBG=fasting blood glucose

Supplementary table 8 shows the total population, number of cases of type 2 diabetes identified during follow-up, and the sensitivity, specificity, and observed risk at different thresholds of risk for model A. For example, using a 10 year risk threshold of 11.1% would identify the top 10% of patients with the highest risk of diabetes using model A. At this threshold, the sensitivity was 45.9%, specificity 90.8%, and observed risk 19.3% (95% confidence interval 19.1% to 19.5%). Using a risk threshold of 6.6% (the top 20%) the corresponding values would be 68.1%, 81.1%, and 14.3%. The thresholds for models B and C will vary according to the strategy chosen for the initial identification of patients using model A so are not presented here.

Figures 3 and 4[Fig f3 f4] are screenshots of the updated web calculator with several clinical examples to show how QDiabetes-2018 could be used within a consultation. Example 1 (figure 3) shows that a white woman aged 40 years with a body mass index of 30 kg/m^2^ and a family history of diabetes has a 10 year estimated risk of type 2 diabetes of 3.6%. If she has polycystic ovary syndrome, her risk is 5.0%. If she also has had gestational diabetes, her risk is 21.1%. If she also has schizophrenia, her risk is 26.5%. If she has a fasting glucose value of 5.5 mmol/L, her 10 year risk is 13.7%. If her fasting glucose was 6.2 mmol/L, her risk would be 67.7%. Example 2 (figure 4) is for a Pakistani man aged 35 who has a body mass index of 30 kg/m^2^. He also has schizophrenia and is prescribed atypical antipsychotics. His 10 year estimated risk of type 2 diabetes is 15.6%. If he is prescribed a statin his 10 year risk is 35.5%. If he has a HBA1c value of 35 mmol/mol, his 10 year risk is 16.8%.

**Figure f3:**
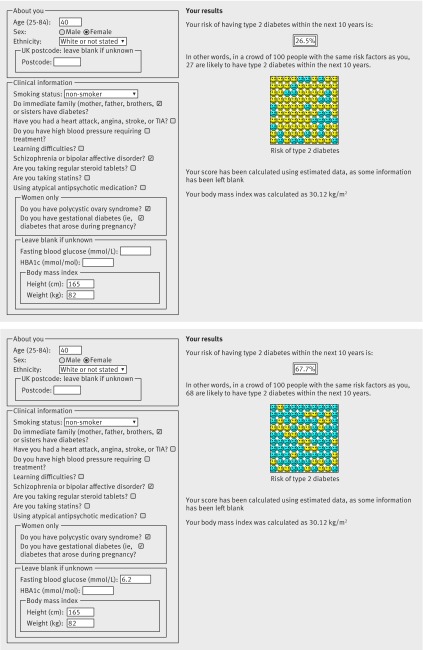
**Fig 3** Clinical example 1: Using QDiabetes to calculate absolute risk of diabetes in a female patient. TIA=transient ischaemic attack

**Figure f4:**
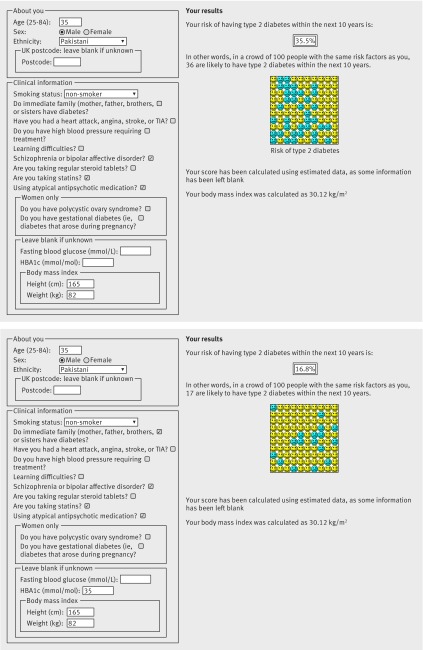
**Fig 4** Clinical example 2: Using QDiabetes to calculate absolute risk of diabetes in a male patient. TIA=transient ischaemic attack

### Decision curve analysis

Figure 5 displays the net benefit curves for men and women. These show that the prediction equations for models A, B, and C had higher net benefit than strategies based on considering either no patients or all patients for intervention across a range of thresholds, and these are useful up to an absolute risk threshold of approximately 40%. Model B had slightly improved net benefit compared with model C and both were better than model A.

**Figure f5:**
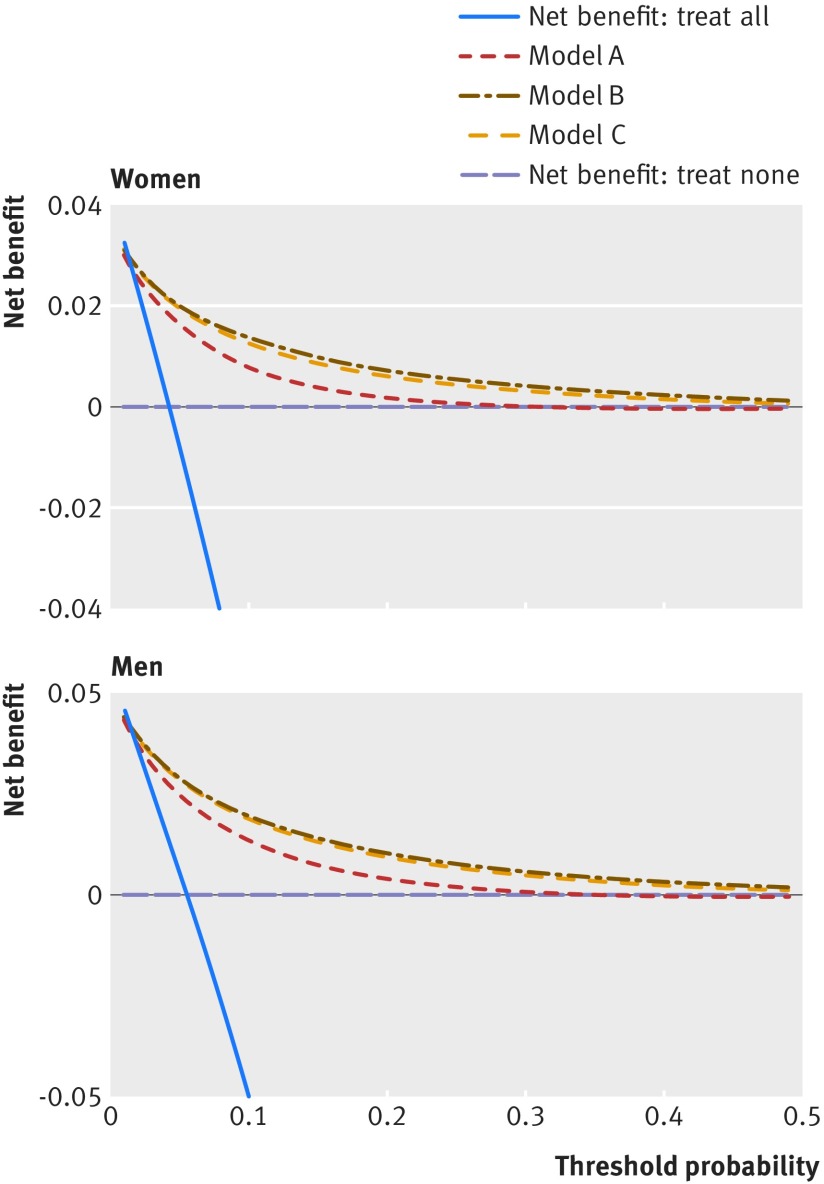
**Fig 5** Net benefit curves for men and women

## Discussion

We have developed and validated updated equations to predict the 10 year risk of type 2 diabetes (QDiabetes-2017) in men and women aged 25 to 84 years. The equations incorporate established predictor variables as well as new risk factors associated with an increased risk of type 2 diabetes. Three models were produced: model A includes existing risk factors (age, ethnicity, deprivation, body mass index, smoking, family history of diabetes in a first degree relative, cardiovascular disease, treated hypertension, and regular use of corticosteroids) and new risk factors (atypical antipsychotics, statins, schizophrenia or bipolar affective disorder, learning disability, gestational diabetes, and polycystic ovary syndrome). The inclusion of these new risk factors will help ensure more accurate estimation of the level of risk in the affected population to improve information for individual patients and for surveillance strategies.

Although the new models are more complex than the existing models, this is unlikely to affect the uptake of the new models as they are all designed to be calculated automatically based on information recorded in the electronic patient record. Figures 3 and 4[Fig f3 f4] included case studies where the impact of additional risk factors for an example patient would lead to different management. For individuals, the presence of these new risk factors could substantially increase their absolute risks of diabetes. These changes in absolute risk could push individuals over a risk threshold, which may then result in different clinical management. However, the actual numbers of people in these groups are comparatively small, so the discrimination statistics are too crude to be able to detect these effects in individuals.

In addition, we have developed two further models, which include blood test results in addition to the risk factors from model A. Model B includes fasting blood glucose and model C includes glycated haemoglobin (HBA1c) as continuous values. This approach improves on current approaches using fixed thresholds for fasting blood glucose or HBA1c,^12^ ^51^ as it takes other risk factors into account and allows a more precise estimation of risk to be communicated to patients. The new models can be used to support the two step approach to the identification of patients at high risk of diabetes, as recommended by NICE guidance^13^ and the NHS Health Checks.^12^ Model A could be used to identify those at high risk of diabetes who require a test for fasting blood glucose or HBA1c. After identifying those patients who meet the criteria for a diagnosis of diabetes, model B could be used to refine the risk estimation in the remaining patients once the result of the fasting blood glucose test is known, since this information provides a more accurate assessment of risk. Similarly, model C could be used when the HBA1c test result is known. Model B had better discrimination and explained more of the variation in time to diagnosis of diabetes than model C. Model B also had the highest sensitivity for identifying cases of diabetes (fig 2), identifying more than two thirds of cases in those determined as being high risk at step 1 compared with the current NHS strategy based on bands of HBA1c that had the lowest sensitivity. Model B also had the highest net benefit, as shown by the decision analysis curve in figure 5, although it was only a small improvement on model C. The strategy based on model B identified a high risk group of 6.7% of the validation cohort, which included nearly half (49.8%) of all patients with a diagnosis of type 2 diabetes over 10 years. Overall, use of model B in strategy 3 and model C in strategy 4 (fig 2) gives more accurate predictions of the future diabetes risk among those tested compared with either strategy 1 or 2 based on blood tests alone.

### Comparisons with the literature

The hazard ratios for the new risk variables included in our final models are similar in both magnitude and direction to those reported in other studies.

*Antipsychotics, mental health problems, and learning disabilities*—Recent published NICE guidance on identification of people at risk of type 2 diabetes highlights the increased risk associated with learning disabilities and mental health problems.^14^ Learning disabilities affected approximately 1% of our derivation cohort and were associated with a 32% increased risk of diabetes in women and 26% increased risk in men at the mean age (model A). This is consistent with other studies^15^ and is likely to be related to adverse lifestyle factors, including lack of exercise.^52^ Schizophrenia or bipolar affective disorder also affected approximately 1% of patients and was associated with a 30% increased risk of diabetes in women and a 26% increased risk in men. Atypical antipsychotics were prescribed for approximately 1% of patients. They were associated with a 74% increased risk of diabetes in women and 52% increased risk in men. This is independent of the risk associated with schizophrenia or bipolar affective disorder, and hence if patients have both factors there will be a compound effect on risk of diabetes. The magnitude of this increased risk was consistent with other studies.^16^ Although the prevalence of each of these conditions is approximately 1%, the magnitude of the effect is substantial and likely to represent an important clinical problem for patients. Clinicians will now be able to use QDiabetes-2018 to provide better information to these patients about both the potential effects of atypical antipsychotics and the interventions to reduce the risk of diabetes.

*Statins*—The increased risk of type 2 diabetes associated with statin use is established. A meta-analysis of 13 statin trials reported a 9% (95% confidence interval 2% to 17%) increased risk.^20^ The risk associated with statin use was higher in our study than in the trials, which may reflect targeting of statins towards those who are already at higher risk of diabetes. Also, the participants in the meta-analysis trials were substantially older (mean age 65 years) than our study participants (mean age 45 years). When similar age groups are compared, the magnitude of the increased risk associated with statins in our study is broadly comparable with that reported in the meta-analysis of clinical trials,^20^ reflecting the interaction between age and statin use. While the magnitude of the diabetes risk associated with statins was of similar magnitude to the increased risk found for atypical antipsychotic drugs, the public health implications may be greater because statins are one of the most commonly prescribed medicines internationally and are targeted at those who already have adverse cardiovascular risk profiles. However, the increased diabetes risk with statins needs to be balanced against the potential reduction in coronary events,^20^ making the provision of accurate information on risks and benefits of statins even more important.

*Gestational diabetes and polycystic ovary syndrome*—We studied two risk factors (gestational diabetes and polycystic ovary syndrome) that only occur in women. Polycystic ovary syndrome is known to be associated with an increased prevalence of diabetes.^53^ It has recently been identified as a risk factor for type 2 diabetes.^54^ ^55^ We found that polycystic ovary syndrome affected 2% of women at baseline, and it was associated with a 41% increased risk in model A. We also found that gestational diabetes was associated with a 4.6-fold increased risk of diabetes, confirming that it is one of the strongest risk factors for the subsequent development of type 2 diabetes.^56^ ^57^ Although recent NICE guidance on diabetes in pregnancy in 2008 and 2015^58^ recommends annual blood glucose testing postnatally for women with a diagnosis of gestational diabetes, only 20% of such women receive regular screening in primary care.^59^ The inclusion of both polycystic ovary syndrome and gestational diabetes in QDiabetes-2018 will ensure the presence of an automated integrated tool available in general practice computer systems to alert clinicians to these patients’ increased risk of diabetes and facilitate proactive follow-up in primary care.

*Comparison with original version of QDiabetes-2009*—Our first QDiabetes model, published in 2009, was based on a cohort followed up between 1993 and 2008. Since then improvements have been made to the QResearch database used to derive the equation, which may have resulted in changes to the model. For example, the number of practices contributing to the database has almost tripled, from 531 in 2009 to 1465 in this study. The size of the derivation cohort has increased threefold, with 178 314 diagnoses of type 2 diabetes arising from 42.7 million person years of observation compared with 78 081 diagnoses of type 2 diabetes arising from 16.4 million person years in 2009.^1^ The recorded prevalence of family history of diabetes has increased by 50%, rising from 9.9% to 14.9%. The baseline prevalence of treated hypertension and corticosteroids have each doubled. The recording of self assigned ethnicity has increased threefold, from 24% to 72.5% in the current study. As a result, we have many more events within the various subgroups. This is reflected in the more precise hazard ratios with tighter confidence intervals and improved performance statistics. Interestingly, the hazard ratios by ethnic group varied between the different models, with models B and C tending to have lower values for non-white ethnic groups compared with model A. The discrimination statistics were, however, broadly similar. Overall, the new models are well calibrated when applied to a separate validation cohort and have high levels of discrimination. Although model A had similar performance to the current QDiabetes model, the other two models showed considerable improvement, with the best overall performance for model B.

### Further methodological considerations

The methods to derive and validate these models are broadly the same as for a range of other clinical risk prediction tools derived from the QResearch database.^1^ ^29^ ^60^ ^61^ ^62^ ^63^ The strengths and limitations of the approach have already been discussed in detail.^1^ ^6^ ^31^ ^62^ ^63^ ^64^ ^65^ In summary, key strengths include size, duration of follow-up, representativeness, and lack of selection, recall, and respondent bias. UK general practices have good levels of accuracy and completeness in recording clinical diagnoses and prescribed drugs.^66^ Our study included approximately 20% of all general practices in England, and the characteristics of the population registered with QResearch are similar to the population registered with other large general practice databases using other clinical computer systems.^5^ It is therefore likely to be representative of the population overall, especially since approximately 98% of the UK population is registered with a general practice. Of all the patients with type 1 or type 2 diabetes excluded at baseline, 9.1% were classified as having type 1 diabetes, which is consistent with other studies using different approaches.^67^ ^68^We think our study has good face validity as it has been conducted in the setting where most patients in the UK are assessed, treated, and followed up.

### Limitations of this study

Limitations of our study include the lack of formal adjudication of diagnoses, information bias, and potential for bias because of missing data. Fractional polynomial terms were identified using complete rather than imputed data. This may have resulted in some bias or less power to detect non-linear trends.^69^ Only 16% of patients had complete data for blood glucose measurements, smoking, and body mass index. However, the characteristics of patients and the magnitude of the hazard ratios on the complete case analysis were broadly similar for both magnitude and direction to the analysis based on imputed data (results shown in supplementary tables 3 and 4), which is reassuring. We used five imputations, which may be fewer than recommended because of practical considerations given the huge size of the dataset. However, given the high degree of missing data for models B and C, additional external validation of these models in datasets with more completely collected data would be valuable before the models are used in clinical practice.

Some under-ascertainment of diagnoses of type 2 diabetes might be present leading to misclassification bias for the outcome. This is because not all patients will have consulted their general practitioner during the study period and been screened for diabetes. Similarly, there may be under-ascertainment of some of the predictor variables such as polycystic ovary syndrome and gestational diabetes, as the baseline prevalence is lower than in other studies.^67^ This may be because gestational diabetes had not been diagnosed or that the diagnosis had not been recorded on the general practice electronic health record. Another limitation is that we have not been able to use oral glucose tolerance testing as a predictor variable as the results of these tests are not stored routinely in the general practice record. We have not taken account of competing risks in this analysis because the results can be counterintuitive^70^ and difficult to use in clinical practice.^71^ However, not accounting for the competing risk of death in elderly patients is likely to result in risk estimates that are too high in this age group.

We excluded patients without a valid deprivation score as this group may represent a more transient population where follow-up could be unreliable or unrepresentative. Their deprivation scores are unlikely to be missing at random so we did not think it would be appropriate to impute them.

Some overfitting might have occurred, but this is unlikely given the large number of events. Generally, to avoid overfitting it is recommended that there are at least 10 events per predictor variable, including the interaction terms.^72^ Our most complex model (model C in women) had 45 predictor variables. Our derivation sample had 178 314 events, giving 3962 events per predictor variable, which is nearly 400 times the minimum recommended level.

The present validation has been done on a randomly selected separate set of practices and individuals to those that were used to develop the score, although the practices all use the same general practice clinical computer system (EMIS, the computer system used by 58% of general practices in England). Some researchers argue that a split sample validation is not necessary when the sample is large enough,^73^ as in our study. Others argue that a split sample validation is still valuable. However, since randomly splitting a huge dataset is likely to result in similar populations, it is preferable to split by time or geographical location to obtain a a non-random selection of practices covering a broader range of settings.^40^ An independent external validation study would be a more stringent test and should be done, but when such studies have examined QDiabetes^6^ ^7^and other risk equations,^65^ ^74^ ^75^ they have shown comparable performance to the validation in the QResearch database.^6^ ^7^ We have published the source code to enable accurate implementation of QDiabetes-2018 on the QDiabetes website (www.qdiabetes.org) alongside earlier versions of the score from previous updates. The rationale for this is to ensure that those interested in reviewing or using the open source will then be able to find the latest available version as the score continues to be updated.

### Conclusions

We have developed updated risk equations (QDiabetes-2018) to quantify absolute risks of type 2 diabetes in people aged 25-84 years, which include established risk factors and new risk factors: atypical antipsychotics, statins, schizophrenia or bipolar affective disorder, learning disability, gestational diabetes, and polycystic ovary syndrome. The updated risk equations provide valid measures of absolute risk in the general population of patients, as shown by the performance in a separate validation cohort. The addition of fasting blood glucose to the updated model (model B) had the best discrimination and sensitivity and potentially improves on currently available risk assessment approaches to identify those at risk of diabetes.

**What is already known on this topic**

Methods to identify those at increased risk of type 2 diabetes are needed to identify patients for whom interventions or more frequent assessment may be required
QDiabetes is currently widely used to estimate 10 year risk of type 2 diabetes in people aged 25-84 years both to communicate risk to patients and to identify patients at high risk for interventions and active surveillance
QDiabetes does not include some well established risk factors and so will underestimate risk in these patients
It also does not include fasting blood glucose or HBA1c values

**What this study adds**

Updated algorithms (QDiabetes-2018) were developed to quantify absolute risks of type 2 diabetes in adults aged 25-84, which include established risk factors and new risk factors such as atypical antipsychotics, statins, schizophrenia or bipolar affective disorder, learning disability, gestational diabetes, and polycystic ovary syndrome, and also can incorporate fasting blood glucose and HBA1c values
The updated risk algorithms provide valid measures of absolute risk in the general population of patients as shown by the performance in a separate validation cohort
The model that includes fasting blood glucose had the best discrimination and the highest sensitivity compared with current recommended practice in the NHS based on bands of either fasting blood glucose or HBA1c

## Web extra

Extra material supplied by authors

## Notes

A simple web calculator (http://qdiabetes.org/2018) can be used to implement the QDiabetes-2018 algorithm. The website also has the open source software for download. We acknowledge the contribution of EMIS practices who contribute to QResearch, and EMIS Health and the University of Nottingham for expertise in establishing, developing, and supporting the QResearch database. The authors acknowledge the financial infrastructure support from the National Institute for Health Research Nottingham Biomedical Research Centre.

Ethical approval: Ethical approval for QResearch is with East Midlands-Derby Research Ethics Committee (reference 03/4/021).

Data sharing: The equations presented in this paper will be released as Open Source Software under the GNU lesser GPL v3. The open source software allows use without charge under the terms of the GNU lesser public license version 3. Closed source software can be licensed at a fee.

Transparency: The lead author (JHC) affirms that the manuscript is an honest, accurate, and transparent account of the study being reported; that no important aspects of the study have been omitted; and that any discrepancies from the study as planned (and, if relevant, registered) have been explained.
