# Supplementary material for: Development and validation of QDiabetes-2018 risk prediction algorithm to estimate future risk of type 2 diabetes: cohort study
Source: BMJ. 2017 Nov 20;359:j5019. doi: 10.1136/bmj.j5019 (PMC5694979; doi:10.1136/bmj.j5019)

1 Supplementary tables

Supplementary table 1a Distribution of risk factors by ethnic group in the derivation cohort. Values are numbers (%) unless otherwise stated.

	White	Indian	Pakistani	Bangladeshi	Other Asian	Caribbean	Black African	Chinese	Other
total population	7136377	188049	101231	81834	122981	80657	179423	65999	230154
men	3547842 (49.7)	97136 (51.7)	56217 (55.5)	46156 (56.4)	57564 (46.8)	36131 (44.8)	85338 (47.6)	27408 (41.5)	108350 (47.1)
BMI recorded	5602202 (78.5)	159442 (84.8)	83533 (82.5)	71948 (87.9)	103174 (83.9)	68091 (84.4)	151521 (84.4)	54629 (82.8)	188151 (81.8)
fasting glucose recorded	1010698 (14.2)	38846 (20.7)	20388 (20.1)	19681 (24.0)	21123 (17.2)	16056 (19.9)	27478 (15.3)	5618 (8.5)	29510 (12.8)
HBA1C recorded	402555 (5.6)	18714 (10.0)	12872 (12.7)	10824 (13.2)	11371 (9.2)	9928 (12.3)	18334 (10.2)	2996 (4.5)	19182 (8.3)
mean age (SD)	45.8 (15.6)	38.4 (12.4)	37.5 (11.7)	35.9 (10.8)	38.2 (11.4)	44.7 (14.0)	38.5 (10.7)	36.0 (11.3)	37.9 (11.1)
mean Townsend score (SD)	0.1 (3.2)	1.5 (2.9)	2.2 (2.5)	4.5 (2.2)	2.1 (2.9)	3.3 (2.6)	3.9 (2.5)	2.5 (3.1)	2.8 (2.9)
mean BMI (SD)	26.0 (5.0)	25.0 (4.4)	26.0 (4.9)	24.9 (4.2)	24.7 (4.4)	27.4 (5.5)	27.2 (5.2)	22.5 (3.6)	25.7 (5.0)
mean fasting glucose (SD)	5.0 (0.6)	5.0 (0.6)	4.9 (0.6)	5.0 (0.7)	5.0 (0.6)	5.0 (0.7)	4.9 (0.6)	4.9 (0.6)	4.9 (0.6)
mean HBA1C (SD)	37.1 (4.5)	38.1 (4.6)	38.3 (4.5)	38.3 (4.7)	37.7 (4.5)	38.4 (4.8)	37.6 (4.7)	37.0 (4.4)	36.9 (4.6)
**smoking recorded**	6806477 (95.4)	184296 (98.0)	99378 (98.2)	80508 (98.4)	120346 (97.9)	79558 (98.6)	175865 (98.0)	64177 (97.2)	224039 (97.3)
Non-smoker	3691699 (51.7)	150585 (80.1)	75210 (74.3)	54063 (66.1)	90654 (73.7)	47643 (59.1)	143317 (79.9)	50837 (77.0)	137787 (59.9)
ex- smoker	1418904 (19.9)	13744 (7.3)	6516 (6.4)	6987 (8.5)	11725 (9.5)	10651 (13.2)	13757 (7.7)	5559 (8.4)	30956 (13.5)
light smoker	975336 (13.7)	15651 (8.3)	11871 (11.7)	12971 (15.9)	12714 (10.3)	15294 (19.0)	13352 (7.4)	5465 (8.3)	35991 (15.6)
moderate smoker	444912 (6.2)	3479 (1.9)	4373 (4.3)	5091 (6.2)	3819 (3.1)	4578 (5.7)	4246 (2.4)	1738 (2.6)	13520 (5.9)
heavy smoker	275626 (3.9)	837 (0.4)	1408 (1.4)	1396 (1.7)	1434 (1.2)	1392 (1.7)	1193 (0.7)	578 (0.9)	5785 (2.5)
**medical characteristics**									
family history of diabetes	950058 (13.3)	58932 (31.3)	31206 (30.8)	31325 (38.3)	30143 (24.5)	24748 (30.7)	31721 (17.7)	10359 (15.7)	50190 (21.8)
treated hypertension	676021 (9.5)	10717 (5.7)	4347 (4.3)	3320 (4.1)	5387 (4.4)	11971 (14.8)	13713 (7.6)	1576 (2.4)	10251 (4.5)
cardiovascular disease	276319 (3.9)	3446 (1.8)	1986 (2.0)	1184 (1.4)	1475 (1.2)	1951 (2.4)	1314 (0.7)	368 (0.6)	2302 (1.0)
bipolar or schizophrenia	52419 (0.7)	973 (0.5)	723 (0.7)	732 (0.9)	621 (0.5)	2018 (2.5)	1691 (0.9)	196 (0.3)	2641 (1.1)

learning disability	51939 (0.7)	529 (0.3)	532 (0.5)	312 (0.4)	291 (0.2)	780 (1.0)	466 (0.3)	126 (0.2)	1117 (0.5)
gestational diabetes	10921 (0.2)	1022 (0.5)	798 (0.8)	1360 (1.7)	764 (0.6)	234 (0.3)	984 (0.5)	229 (0.3)	902 (0.4)
polycystic ovaries	65798 (0.9)	3528 (1.9)	1784 (1.8)	1514 (1.9)	1708 (1.4)	1259 (1.6)	1796 (1.0)	429 (0.7)	3348 (1.5)
**Medication**									
statins	489078 (6.9)	8761 (4.7)	4700 (4.6)	3852 (4.7)	4476 (3.6)	4853 (6.0)	4233 (2.4)	1018 (1.5)	5998 (2.6)
atypical antipsychotics	50791 (0.7)	851 (0.5)	731 (0.7)	715 (0.9)	690 (0.6)	1232 (1.5)	1369 (0.8)	128 (0.2)	2148 (0.9)
corticosteroids	223952 (3.1)	3448 (1.8)	2299 (2.3)	1180 (1.4)	1612 (1.3)	1806 (2.2)	1417 (0.8)	346 (0.5)	2623 (1.1)

Supplementary table 1b characteristics of patients with either fasting blood glucose or HBA1C recorded compared with those who have neither recorded.

	**no FBS or HBA1C recorded**	**FBS or HBA1C recorded**
total population	6681086	1505619
men	3360238 (50.3)	701904 (46.6)
BMI recorded	5113502 (76.5)	1369189 (90.9)
fasting blood glucose recorded	n/a	1189398 (79.0)
HBA1C recorded	n/a	506776 (33.7)
mean age (SD)	43.0 (14.7)	53.0 (15.6)
mean Townsend score (SD)	0.5 (3.3)	0.3 (3.2)
mean BMI (SD)	25.5 (4.8)	27.5 (5.4)
**ethnicity recorded**	4690461 (70.2)	1243087 (82.6)
White/not recorded	5878090 (88.0)	1258287 (83.6)
Indian	138533 (2.1)	49516 (3.3)
Pakistani	72580 (1.1)	28651 (1.9)
Bangladeshi	55365 (0.8)	26469 (1.8)
Other Asian	94936 (1.4)	28045 (1.9)
Caribbean	57798 (0.9)	22859 (1.5)
Black African	138566 (2.1)	40857 (2.7)
Chinese	58244 (0.9)	7755 (0.5)
Other	186974 (2.8)	43180 (2.9)
**smoking recorded**	6335946 (94.8)	1498698 (99.5)
non-smoker	3594057 (53.8)	847738 (56.3)
ex-smoker	1156694 (17.3)	362105 (24.1)
light smoker	930197 (13.9)	168448 (11.2)
moderate smoker	412550 (6.2)	73206 (4.9)
heavy smoker	242448 (3.6)	47201 (3.1)
**medical characteristics**		
family history of diabetes	922953 (13.8)	295729 (19.6)
treated hypertension	383136 (5.7)	354167 (23.5)
cardiovascular disease	145587 (2.2)	144758 (9.6)
bipolar affective disorder or schizophrenia	42711 (0.6)	19303 (1.3)
learning disability	42383 (0.6)	13709 (0.9)
gestational diabetes	8618 (0.1)	8596 (0.6)
polycystic ovaries	59092 (0.9)	22072 (1.5)
**medication**		
statins	235929 (3.5)	291040 (19.3)
atypical antipsychotics	38984 (0.6)	19671 (1.3)
corticosteroids	165669 (2.5)	73014 (4.8)

Supplementary table 2 Incident cases of type 2 diabetes by ethnic group in the derivation and validation cohorts.

	Derivation cohort		Validation cohort	
	**Incident cases of type 2 diabetes**	**Person years (in 1000's)**	**Incident cases of type 2 diabetes**	**Person years (in 1000's)**
**total**	**178,314**	**42,718**	**62,326**	**14,317**
women	77,895	21,561	27,311	7,242
men	100,419	21,157	35,015	7,076
**Ethnic group**				
White/not recorded	149,948	38,475	53,431	13,059
Indian	6,181	795	1,866	233
Pakistani	4,329	443	1,676	164
Bangladeshi	3,949	365	1,097	93
Other Asian	2,998	461	1,065	146
Caribbean	3,499	428	1,056	127
Black African	3,448	681	929	186
Chinese	590	222	163	55
Other	3,372	849	1,043	253

Supplementary Table 3: Adjusted hazard ratios (95% CI) for type 2 diabetes in women for models A, B and C from complete case analysis. For fractional polynomial terms & age interactions see footnotes and figures.

	**Model A Adjusted hazard ratio (95% CI)**	**Model B Adjusted hazard ratio (95% CI)**	**Model C Adjusted hazard ratio (95% CI)**
Townsend Score (5-unit increase)	1.18 (1.16 to 1.19)	1.15 (1.12 to 1.17)	1.07 (1.03 to 1.12)
**Ethnic group**			
White or not recorded	1.00	1.00	1.00
Indian	2.95 (2.83 to 3.08)	2.32 (2.17 to 2.49)	1.02 (0.90 to 1.16)
Pakistani	3.87 (3.68 to 4.07)	2.90 (2.66 to 3.16)	1.18 (1.03 to 1.35)
Bangladeshi	6.37 (6.05 to 6.71)	3.94 (3.64 to 4.27)	1.61 (1.39 to 1.87)
Other Asian	3.15 (2.97 to 3.34)	2.28 (2.07 to 2.52)	1.30 (1.11 to 1.53)
Caribbean	1.54 (1.47 to 1.62)	1.56 (1.43 to 1.69)	0.94 (0.82 to 1.08)
Black African	1.35 (1.27 to 1.42)	1.41 (1.28 to 1.56)	0.77 (0.65 to 0.91)
Chinese	2.61 (2.30 to 2.95)	2.17 (1.75 to 2.69)	1.27 (0.84 to 1.91)
Other	1.48 (1.40 to 1.56)	1.43 (1.30 to 1.57)	0.75 (0.64 to 0.88)
**smoking status**			
non-smoker	1.00	1.00	1.00
ex-smoker	1.07 (1.05 to 1.10)	1.04 (1.01 to 1.08)	0.97 (0.91 to 1.04)
light smoker	1.37 (1.33 to 1.40)	1.23 (1.17 to 1.30)	0.97 (0.89 to 1.07)
moderate smoker	1.48 (1.43 to 1.54)	1.40 (1.31 to 1.50)	1.00 (0.88 to 1.14)
heavy smoker	1.74 (1.68 to 1.82)	1.40 (1.29 to 1.52)	1.22 (1.06 to 1.42)
**Medical characteristics‡**			
family history of diabetes†	1.68 (1.64 to 1.72)	1.48 (1.42 to 1.54)	1.44 (1.34 to 1.55)
treated hypertension	1.55 (1.52 to 1.58)	1.21 (1.17 to 1.25)	1.38 (1.30 to 1.46)
cardiovascular disease	1.17 (1.13 to 1.21)	1.12 (1.07 to 1.17)	1.05 (0.96 to 1.13)
bipolar or schizophrenia	1.30 (1.21 to 1.40)	1.11 (0.97 to 1.28)	1.38 (1.11 to 1.70)
learning disability†	1.29 (1.16 to 1.45)	1.29 (1.02 to 1.63)	0.75 (0.51 to 1.10)
gestational diabetes	4.64 (4.35 to 4.95)	2.62 (2.36 to 2.92)	2.20 (1.90 to 2.54)
polycystic ovaries	1.39 (1.30 to 1.47)	1.28 (1.15 to 1.42)	1.23 (1.04 to 1.45)
**current medication‡**			
statins†	1.91 (1.82 to 2.01)	1.64 (1.52 to 1.76)	1.52 (1.34 to 1.73)
atypical antipsychotics†	1.68 (1.54 to 1.84)	1.39 (1.17 to 1.64)	1.46 (1.13 to 1.88)
corticosteroids	1.31 (1.28 to 1.34)	1.39 (1.33 to 1.45)	1.19 (1.10 to 1.28)

†interaction with age; hazard ratio evaluated at mean age
‡compared with patients without this characteristic

Model A includes fractional polynomial terms for age ($age^{0.5}$, age^{3}) and BMI (BMI, BMI^{3}) with interaction terms between age and atypical antipsychotics, statins, learning disability, body mass index, family history of diabetes
Model B same as Model A plus fractional polynomial terms for fasting blood glucose (fasting $glucose^{-1}$, fasting $glucose^{-1}$ log (fasting glucose)) and interaction terms between age and fasting glucose
Model C same as Model A plus fractional polynomial terms for HBA1C ($HBA1C^{0.5}$, HBA1C) and interaction terms between age and HBA1C

Supplementary table 4: Adjusted hazard ratios (95% CI) for type 2 diabetes in men for models A, B and C from complete case analysis. For fractional polynomial terms & interactions see footnotes and figures

	Model A Adjusted hazard ratio (95% CI)	Model B Adjusted hazard ratio (95% CI)	Model C Adjusted hazard ratio (95% CI)
Townsend Score (5-unit increase)	1.15 (1.13 to 1.16)	1.13 (1.10 to 1.15)	1.01 (0.98 to 1.06)
**Ethnic group**			
White or not recorded	1.00	1.00	1.00
Indian	2.91 (2.80 to 3.03)	2.23 (2.09 to 2.37)	1.10 (0.98 to 1.23)
Pakistani	3.43 (3.28 to 3.60)	2.92 (2.68 to 3.17)	1.33 (1.16 to 1.52)
Bangladeshi	5.23 (4.98 to 5.50)	3.61 (3.32 to 3.91)	1.37 (1.16 to 1.62)
Other Asian	3.04 (2.88 to 3.21)	2.55 (2.33 to 2.79)	1.40 (1.21 to 1.63)
Caribbean	1.52 (1.44 to 1.61)	1.54 (1.40 to 1.70)	0.85 (0.73 to 1.00)
Black African	1.93 (1.84 to 2.04)	1.98 (1.80 to 2.17)	0.88 (0.74 to 1.04)
Chinese	1.93 (1.70 to 2.19)	1.83 (1.45 to 2.31)	1.22 (0.82 to 1.83)
Other	1.47 (1.40 to 1.55)	1.44 (1.30 to 1.59)	0.88 (0.75 to 1.04)
**smoking status**			
non-smoker	1.00	1.00	1.00
ex-smoker	1.17 (1.15 to 1.19)	1.07 (1.04 to 1.10)	1.02 (0.96 to 1.07)
light smoker	1.38 (1.35 to 1.41)	1.30 (1.25 to 1.36)	0.93 (0.86 to 1.01)
moderate smoker	1.39 (1.34 to 1.43)	1.39 (1.30 to 1.49)	0.91 (0.81 to 1.03)
heavy smoker	1.57 (1.52 to 1.62)	1.48 (1.38 to 1.58)	0.84 (0.73 to 0.96)
**medical conditions‡**			
family history of diabetes†	1.80 (1.76 to 1.83)	1.54 (1.47 to 1.60)	1.52 (1.41 to 1.64)
treated hypertension	1.36 (1.34 to 1.39)	1.20 (1.17 to 1.23)	1.29 (1.22 to 1.35)
cardiovascular disease	1.21 (1.18 to 1.24)	1.17 (1.13 to 1.22)	1.19 (1.11 to 1.27)
bipolar or schizophrenia	1.23 (1.15 to 1.32)	1.21 (1.05 to 1.38)	1.15 (0.93 to 1.44)
learning disability†	1.22 (1.11 to 1.35)	1.34 (1.09 to 1.63)	1.07 (0.79 to 1.45)
**Current medication‡**			
statins†	1.75 (1.69 to 1.81)	1.48 (1.40 to 1.56)	1.47 (1.33 to 1.62)
atypical antipsychotics†	1.50 (1.37 to 1.64)	1.35 (1.14 to 1.59)	1.00 (0.76 to 1.31)
corticosteroids	1.23 (1.19 to 1.26)	1.30 (1.24 to 1.37)	1.07 (0.98 to 1.17)

†interaction with age; hazard ratio evaluated at mean age
‡compared with patients without this characteristic
Model A includes fractional polynomial terms for age (ln(age), age^3) and BMI (BMI^2, BMI^3) and interactions between age and atypical antipsychotics, statins, learning disability, body mass index, family history of diabetes
Model B is same as model A plus fractional polynomial terms for fasting blood glucose (fasting $glucose^{-0.5}$, fasting $glucose^{-0.5}$ log (fasting glucose)) and interactions between age and fasting glucose
Model C is same as model A plus fractional polynomial terms for HBA1C ($HBA1C^{0.5}$, HBA1C) and interactions between age and HBA1C

Supplementary table 5 performance of model A and B for subgroups of women from the validation cohort.

Subgroup		Model A	Model B
< 40 years	D statistic	2.54 (2.47 to 2.60)	3.03 (2.96 to 3.10)
< 40 years	Harrell's C	.877 (.869 to .885)	.914 (.908 to .921)
< 40 years	R^2	60.6 (59.4 to 61.8)	68.7 (67.7 to 69.6)
40-59 years	D statistic	1.98 (1.95 to 2.01)	2.59 (2.54 to 2.63)
40-59 years	Harrell's C	.824 (.82 to .828)	.88 (.877 to .883)
40-59 years	R^2	48.4 (47.6 to 49.2)	61.5 (60.7 to 62.3)
60 + years	D statistic	1.28 (1.25 to 1.31)	2.09 (2.04 to 2.14)
60 + years	Harrell's C	.728 (.724 to .733)	.83 (.825 to .835)
60 + years	R^2	28.2 (27.3 to 29.1)	51.1 (49.9 to 52.3)
atypical antipsychotics	D statistic	1.35 (1.19 to 1.51)	2.09 (1.89 to 2.3)
atypical antipsychotics	Harrell's C	.743 (.72 to .765)	.787 (.744 to .831)
atypical antipsychotics	R^2	30.3 (25.4 to 35.2)	51.1 (46.3 to 56)
corticosteroids	D statistic	1.5 (1.44 to 1.56)	2.15 (2.08 to 2.23)
corticosteroids	Harrell's C	.764 (.755 to .773)	.804 (.788 to .82)
corticosteroids	R^2	35 (33.2 to 36.9)	52.5 (50.8 to 54.2)
cardiovascular disease	D statistic	1.01 (.943 to 1.08)	1.88 (1.78 to 1.97)
cardiovascular disease	Harrell's C	.681 (.67 to .692)	.808 (.795 to .822)
cardiovascular disease	R^2	19.5 (17.5 to 21.6)	45.7 (43.2 to 48.2)
learning disability	D statistic	1.52 (1.28 to 1.77)	2.24 (1.92 to 2.56)
learning disability	Harrell's C	.769 (.733 to .805)	.79 (.703 to .877)
learning disability	R^2	35.7 (28.2 to 43.2)	54.6 (47.5 to 61.7)
bipolar affective disorder or schizophrenia	D statistic	1.45 (1.29 to 1.62)	2.17 (1.95 to 2.39)
bipolar affective disorder or schizophrenia	Harrell's C	.748 (.722 to .775)	.791 (.745 to .836)
bipolar affective disorder or schizophrenia	R^2	33.5 (28.4 to 38.7)	53 (47.9 to 58)
statins	D statistic	1.1 (1.06 to 1.15)	1.89 (1.82 to 1.96)
statins	Harrell's C	.697 (.69 to .705)	.808 (.797 to .819)
statins	R^2	22.5 (21.1 to 24)	46 (44.1 to 48)
treated hypertension	D statistic	1.11 (1.07 to 1.14)	1.92 (1.87 to 1.96)
treated hypertension	Harrell's C	.696 (.691 to .702)	.807 (.799 to .814)
treated hypertension	R^2	22.6 (21.6 to 23.7)	46.8 (45.6 to 47.9)
White or not recorded	D statistic	2.05 (2.03 to 2.08)	2.69 (2.65 to 2.74)
White or not recorded	Harrell's C	.833 (.831 to .836)	.889 (.887 to .891)

White or not recorded	R^2	50.1 (49.6 to 50.7)	63.4 (62.6 to 64.2)
Indian	D statistic	1.77 (1.65 to 1.89)	2.36 (2.21 to 2.51)
Indian	Harrell's C	.813 (.799 to .826)	.868 (.856 to .88)
Indian	R^2	42.8 (39.4 to 46.2)	57 (53.9 to 60.1)
Pakistani	D statistic	1.66 (1.54 to 1.78)	2.28 (2.13 to 2.42)
Pakistani	Harrell's C	.806 (.793 to .82)	.863 (.852 to .875)
Pakistani	R^2	39.7 (36.2 to 43.2)	55.3 (52.2 to 58.4)
Bangladeshi	D statistic	1.52 (1.37 to 1.67)	1.99 (1.83 to 2.15)
Bangladeshi	Harrell's C	.776 (.757 to .795)	.839 (.821 to .858)
Bangladeshi	R^2	35.6 (30.9 to 40.2)	48.6 (44.7 to 52.5)
Other Asian	D statistic	1.74 (1.58 to 1.9)	2.47 (2.28 to 2.67)
Other Asian	Harrell's C	.81 (.791 to .829)	.87 (.852 to .888)
Other Asian	R^2	42 (37.4 to 46.5)	59.4 (55.5 to 63.2)
Caribbean	D statistic	1.61 (1.47 to 1.75)	2.39 (2.18 to 2.6)
Caribbean	Harrell's C	.789 (.773 to .805)	.865 (.847 to .883)
Caribbean	R^2	38.3 (34.2 to 42.4)	57.7 (53.4 to 62.1)
Black African	D statistic	1.58 (1.42 to 1.75)	2.37 (2.16 to 2.57)
Black African	Harrell's C	.792 (.767 to .818)	.869 (.847 to .89)
Black African	R^2	37.5 (32.5 to 42.4)	57.2 (52.9 to 61.5)
Chinese	D statistic	2.45 (2.04 to 2.85)	2.99 (2.41 to 3.57)
Chinese	Harrell's C	.872 (.829 to .915)	.912 (.871 to .953)
Chinese	R^2	58.8 (50.7 to 66.9)	68 (59.6 to 76.5)
Other	D statistic	1.98 (1.82 to 2.13)	2.65 (2.47 to 2.83)
Other	Harrell's C	.837 (.82 to .854)	.891 (.876 to .906)
Other	R^2	48.3 (44.4 to 52.1)	62.6 (59.4 to 65.8)
family history of diabetes	D statistic	1.85 (1.81 to 1.89)	2.47 (2.41 to 2.53)
family history of diabetes	Harrell's C	.808 (.803 to .812)	.835 (.828 to .843)
family history of diabetes	R^2	45 (44 to 46)	59.3 (58.2 to 60.4)
overall	D statistic	2.07 (2.05 to 2.09)	2.69 (2.65 to 2.73)
overall	Harrell's C	.834 (.832 to .837)	.889 (.887 to .891)
overall	R^2	50.5 (50 to 51)	63.3 (62.7 to 64)

Supplementary table 6a performance of model A and B for subgroups of men from the validation cohort.

Subgroup		Model A	Model B
< 40 years	D statistic	2.18 (2.12 to 2.23)	2.62 (2.54 to 2.69)
< 40 years	Harrell's C	.843 (.835 to .851)	.884 (.877 to .892)
< 40 years	R^2	53.1 (51.8 to 54.4)	62 (60.7 to 63.4)
40-59 years	D statistic	1.71 (1.69 to 1.74)	2.23 (2.2 to 2.27)
40-59 years	Harrell's C	.785 (.781 to .788)	.845 (.841 to .849)
40-59 years	R^2	41.1 (40.4 to 41.8)	54.3 (53.6 to 55.1)
60 + years	D statistic	1.19 (1.16 to 1.22)	1.87 (1.82 to 1.91)
60 + years	Harrell's C	.709 (.704 to .713)	.803 (.799 to .807)
60 + years	R^2	25.3 (24.5 to 26.2)	45.5 (44.3 to 46.7)
atypical antipsychotics	D statistic	1.28 (1.12 to 1.44)	1.81 (1.63 to 1.99)
atypical antipsychotics	Harrell's C	.748 (.721 to .774)	.777 (.713 to .84)
atypical antipsychotics	R^2	28.1 (23 to 33.2)	43.9 (39.1 to 48.7)
corticosteroids	D statistic	1.43 (1.36 to 1.5)	1.98 (1.89 to 2.06)
corticosteroids	Harrell's C	.751 (.74 to .761)	.782 (.762 to .801)
corticosteroids	R^2	32.8 (30.6 to 35)	48.3 (46.2 to 50.4)
cardiovascular disease	D statistic	1.09 (1.04 to 1.14)	1.74 (1.68 to 1.8)
cardiovascular disease	Harrell's C	.688 (.68 to .696)	.787 (.777 to .797)
cardiovascular disease	R^2	22 (20.5 to 23.5)	42 (40.4 to 43.6)
learning disability	D statistic	1.65 (1.43 to 1.87)	2.1 (1.78 to 2.42)
learning disability	Harrell's C	.785 (.752 to .818)	.819 (.758 to .88)
learning disability	R^2	39.3 (33 to 45.6)	51.2 (43.6 to 58.8)
bipolar affective disorder or schizophrenia	D statistic	1.33 (1.17 to 1.48)	1.88 (1.69 to 2.07)
bipolar affective disorder or schizophrenia	Harrell's C	.745 (.723 to .768)	.774 (.718 to .831)
bipolar affective disorder or schizophrenia	R^2	29.6 (24.7 to 34.5)	45.7 (40.8 to 50.7)
statins	D statistic	1.08 (1.04 to 1.11)	1.71 (1.66 to 1.76)
statins	Harrell's C	.689 (.683 to .695)	.787 (.779 to .795)
statins	R^2	21.7 (20.5 to 22.9)	41.1 (39.6 to 42.7)
treated hypertension	D statistic	1.06 (1.02 to 1.09)	1.72 (1.67 to 1.76)
treated hypertension	Harrell's C	.685 (.679 to .691)	.785 (.777 to .792)
treated hypertension	R^2	21 (19.9 to 22.1)	41.3 (40.1 to 42.5)
White or not recorded	D statistic	1.92 (1.9 to 1.94)	2.45 (2.42 to 2.47)
White or not recorded	Harrell's C	.814 (.812 to .817)	.866 (.864 to .869)

White or not recorded	R^2	46.9 (46.4 to 47.4)	58.8 (58.2 to 59.4)
Indian	D statistic	1.58 (1.48 to 1.69)	2.11 (1.98 to 2.23)
Indian	Harrell's C	.788 (.774 to .801)	.845 (.832 to .859)
Indian	R^2	37.5 (34.3 to 40.6)	51.4 (48.4 to 54.5)
Pakistani	D statistic	1.54 (1.42 to 1.65)	2.05 (1.92 to 2.18)
Pakistani	Harrell's C	.779 (.764 to .795)	.837 (.818 to .857)
Pakistani	R^2	36 (32.5 to 39.5)	50.1 (46.9 to 53.3)
Bangladeshi	D statistic	1.53 (1.39 to 1.67)	1.97 (1.82 to 2.11)
Bangladeshi	Harrell's C	.776 (.757 to .795)	.835 (.819 to .851)
Bangladeshi	R^2	35.9 (31.6 to 40.1)	48 (44.3 to 51.7)
Other Asian	D statistic	1.51 (1.37 to 1.65)	1.97 (1.83 to 2.12)
Other Asian	Harrell's C	.791 (.775 to .808)	.846 (.828 to .864)
Other Asian	R^2	35.2 (30.9 to 39.5)	48.2 (44.6 to 51.8)
Caribbean	D statistic	1.62 (1.47 to 1.77)	2.08 (1.91 to 2.25)
Caribbean	Harrell's C	.779 (.76 to .799)	.838 (.819 to .857)
Caribbean	R^2	38.5 (34.1 to 42.9)	50.7 (46.7 to 54.8)
Black African	D statistic	1.53 (1.38 to 1.68)	2.14 (1.99 to 2.3)
Black African	Harrell's C	.774 (.753 to .795)	.847 (.828 to .867)
Black African	R^2	35.8 (31.2 to 40.3)	52.3 (48.8 to 55.9)
Chinese	D statistic	1.96 (1.55 to 2.37)	2.43 (2 to 2.86)
Chinese	Harrell's C	.847 (.805 to .89)	.9 (.858 to .941)
Chinese	R^2	47.8 (37.3 to 58.4)	58.5 (49.9 to 67.2)
Other ethnic group	D statistic	1.88 (1.74 to 2.03)	2.36 (2.18 to 2.54)
Other ethnic group	Harrell's C	.827 (.809 to .845)	.877 (.858 to .896)
Other ethnic group	R^2	45.9 (42 to 49.7)	57.1 (53.2 to 60.9)
family history of diabetes	D statistic	1.6 (1.56 to 1.64)	2.13 (2.09 to 2.17)
family history of diabetes	Harrell's C	.773 (.768 to .779)	.807 (.797 to .816)
family history of diabetes	R^2	38 (36.8 to 39.2)	52 (51.1 to 52.9)
overall	D statistic	1.91 (1.89 to 1.93)	2.42 (2.4 to 2.45)
overall	Harrell's C	.814 (.812 to .816)	.866 (.863 to .868)
overall	R^2	46.6 (46.1 to 47.1)	58.4 (57.9 to 58.8)

Supplementary table 7 calibration slope overall and by sub-group for model A and model B in men and women in the validation cohort.

	**women**		**Men**	
	**Slope (95% CI)**	**Slope (95% CI)**	**Slope (95% CI)**	**Slope (95% CI)**
group	**Model A**	**Model B**	**Model A**	**Model B**
**overall**	0.997 (0.986 to 1.008)	0.993 (0.978 to 1.007)	0.986 (0.976 to 0.996)	0.985 (0.975 to 0.996)
< 40 years	1.033 (1.007 to 1.058)	1.018 (0.988 to 1.048)	1.001 (0.976 to 1.027)	1.004 (0.976 to 1.031)
< 40-59 years	1.025 (1.008 to 1.041)	1.012 (0.995 to 1.029)	1.003 (0.989 to 1.018)	0.997 (0.983 to 1.011)
< 60+ years	0.935 (0.915 to 0.955)	0.963 (0.942 to 0.985)	0.950 (0.929 to 0.971)	0.963 (0.941 to 0.985)
**ethnicity**				
White	1.059 (1.046 to 1.073)	1.031 (1.015 to 1.047)	1.040 (1.027 to 1.052)	1.021 (1.008 to 1.033)
Indian	0.777 (0.719 to 0.834)	0.812 (0.758 to 0.867)	0.741 (0.689 to 0.793)	0.796 (0.745 to 0.847)
Pakistani	0.725 (0.670 to 0.780)	0.775 (0.722 to 0.828)	0.691 (0.637 to 0.745)	0.752 (0.706 to 0.798)
Bangladeshi	0.636 (0.570 to 0.703)	0.644 (0.592 to 0.696)	0.731 (0.663 to 0.799)	0.739 (0.687 to 0.791)
Other Asian	0.744 (0.673 to 0.816)	0.836 (0.766 to 0.905)	0.715 (0.646 to 0.783)	0.754 (0.699 to 0.808)
Caribbean	0.821 (0.743 to 0.899)	0.876 (0.806 to 0.947)	0.867 (0.781 to 0.954)	0.843 (0.773 to 0.912)
Black African	0.731 (0.648 to 0.814)	0.846 (0.773 to 0.919)	0.735 (0.659 to 0.812)	0.844 (0.783 to 0.905)
Chinese	1.057 (0.872 to 1.243)	1.017 (0.835 to 1.199)	0.968 (0.757 to 1.179)	0.948 (0.788 to 1.108)
Other	0.858 (0.787 to 0.929)	0.916 (0.855 to 0.978)	0.887 (0.815 to 0.960)	0.901 (0.837 to 0.965)
**medical characteristics**				
family history of diabetes	0.886 (0.866 to 0.905)	0.905 (0.885 to 0.925)	0.860 (0.838 to 0.883)	0.889 (0.872 to 0.907)
cardiovascular disease	0.748 (0.699 to 0.797)	0.847 (0.810 to 0.884)	0.840 (0.804 to 0.877)	0.877 (0.848 to 0.905)
learning disability	0.758 (0.625 to 0.890)	0.845 (0.729 to 0.961)	0.769 (0.662 to 0.876)	0.804 (0.686 to 0.921)
bipolar affective disorder or schizophrenia	0.754 (0.661 to 0.846)	0.838 (0.755 to 0.921)	0.676 (0.593 to 0.758)	0.763 (0.686 to 0.840)
treated hypertension	0.817 (0.793 to 0.842)	0.885 (0.863 to 0.907)	0.821 (0.795 to 0.848)	0.878 (0.856 to 0.899)
**medication**				
atypical antipsychotics	0.731 (0.643 to 0.818)	0.833 (0.759 to 0.907)	0.672 (0.586 to 0.757)	0.745 (0.671 to 0.818)
corticosteroids	0.871 (0.834 to 0.909)	0.877 (0.847 to 0.907)	0.880 (0.833 to 0.927)	0.887 (0.850 to 0.924)
statins	0.796 (0.764 to 0.828)	0.859 (0.826 to 0.892)	0.825 (0.797 to 0.853)	0.868 (0.842 to 0.894)

Supplementary table 8 sensitivity, specificity, observed risk (95% confidence intervals) of type 2 diabetes for 2,629,940 patients in the validation cohort based on Model A.

Top centile	Threshold 10-year diabetes risk using Model A (%)	Total incident cases	True negative	False negative	False positive	True positive	Sensitivity (%)	Specificity (%)	Observed risk (%)	Lower CI	Upper CI
top 10%	11.1	58,130	2,335,487	31,459	236,323	26,671	45.9	90.8	19.3	19.1	19.5
top 20%	6.6	58,130	2,085,391	18,561	486,419	39,569	68.1	81.1	14.3	14.2	14.5
top 30%	4.4	58,130	1,830,089	10,869	741,721	47,261	81.3	71.2	11.4	11.3	11.5
top 40%	2.9	58,130	1,571,741	6,223	1,000,069	51,907	89.3	61.1	9.5	9.4	9.6
top 50%	1.9	58,130	1,311,601	3,369	1,260,209	54,761	94.2	51.0	8.1	8.0	8.1

Supplementary Figure S1a

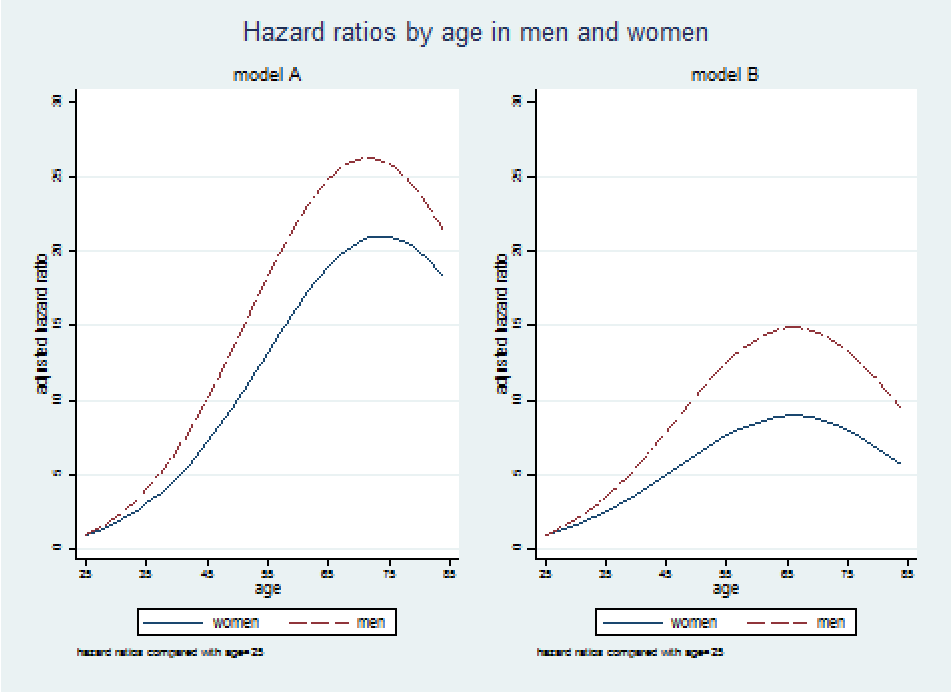

Supplementary Figure S1b

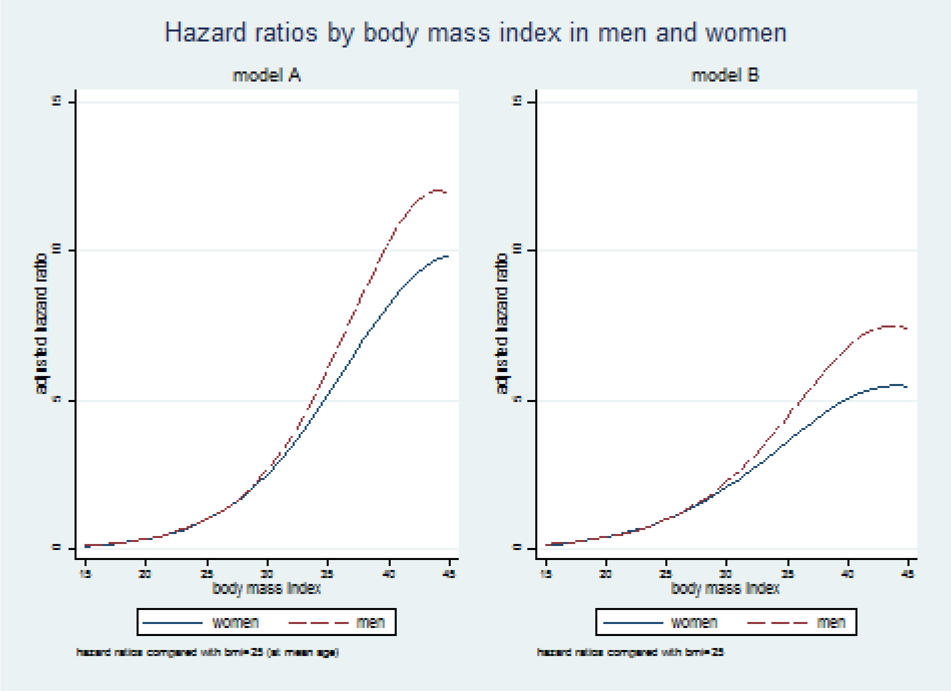

Supplementary Figure S1c

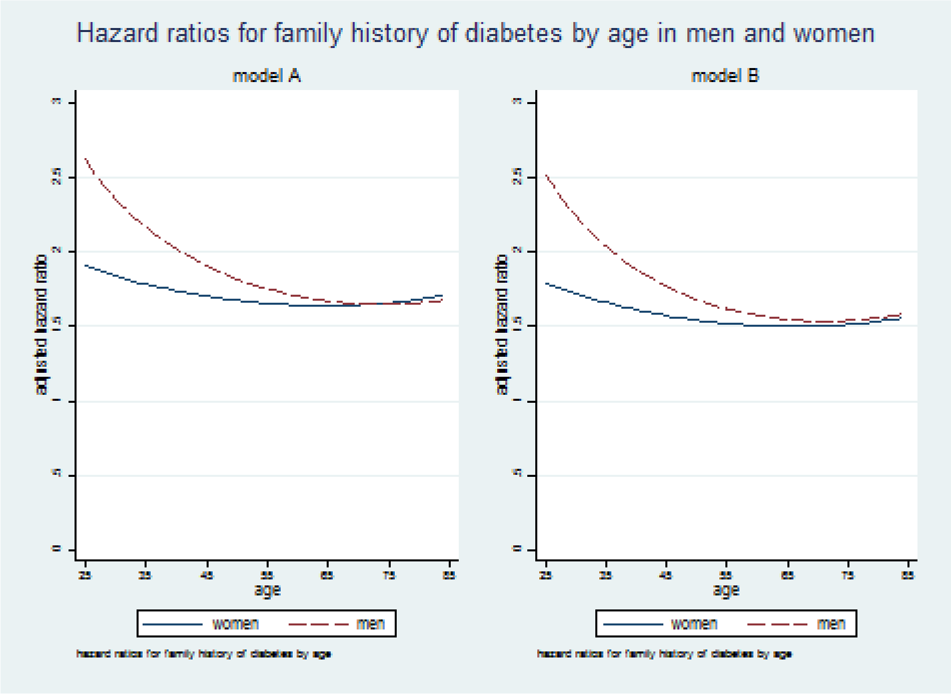

Supplementary Figure S1d

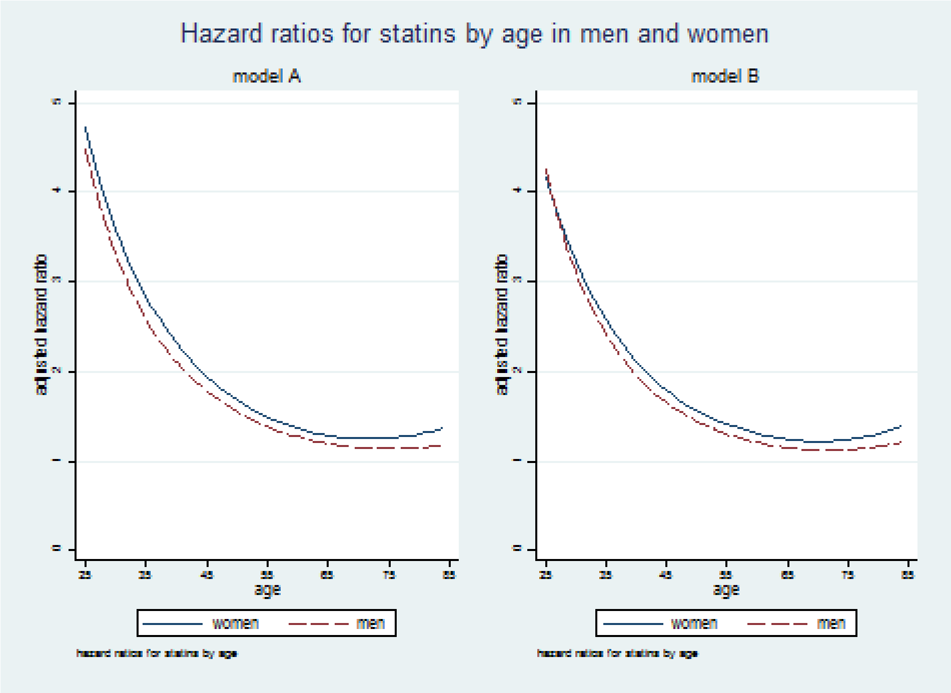

Supplementary Figure S1e

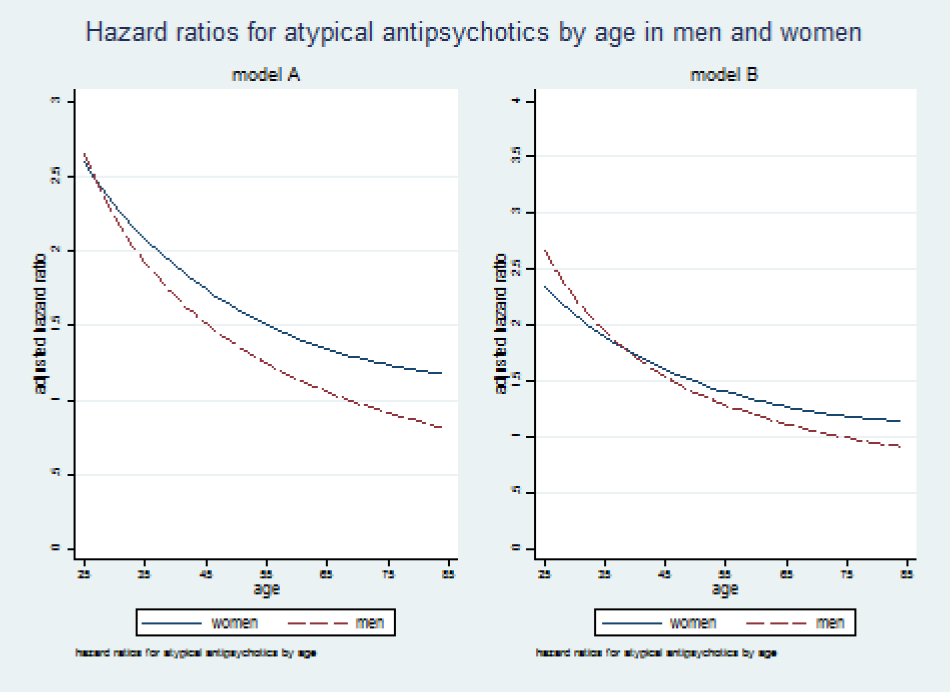

Supplementary Figure S1f

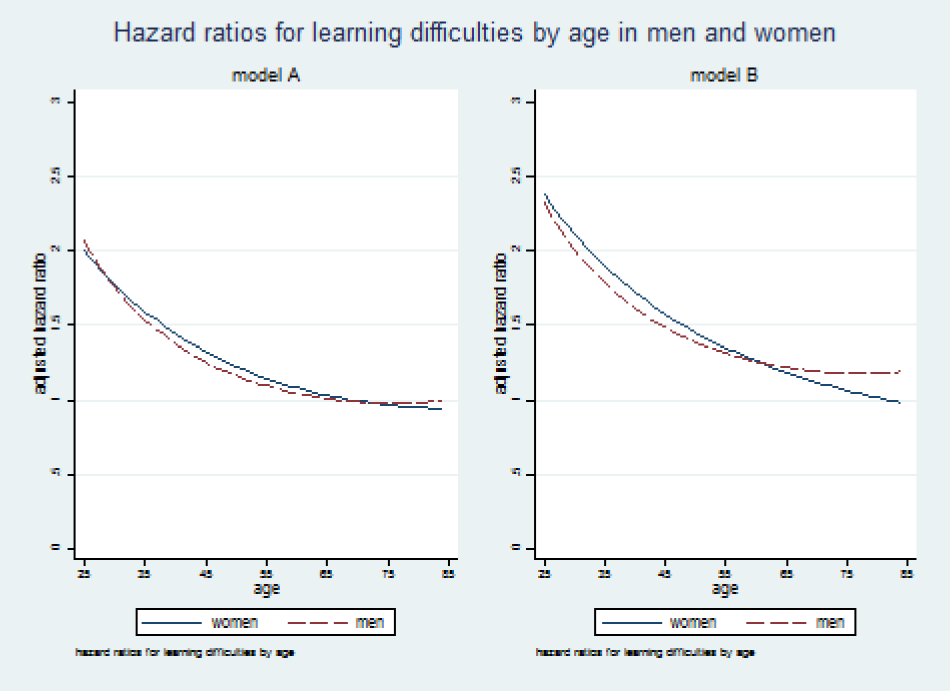

Supplementary Figure S1g

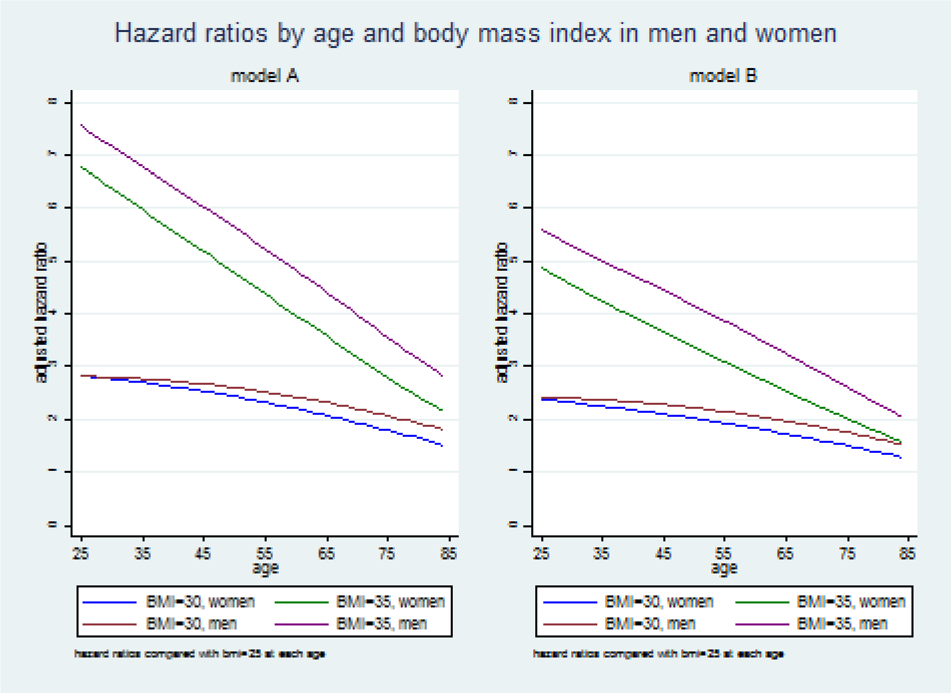

Supplementary Figure S1h

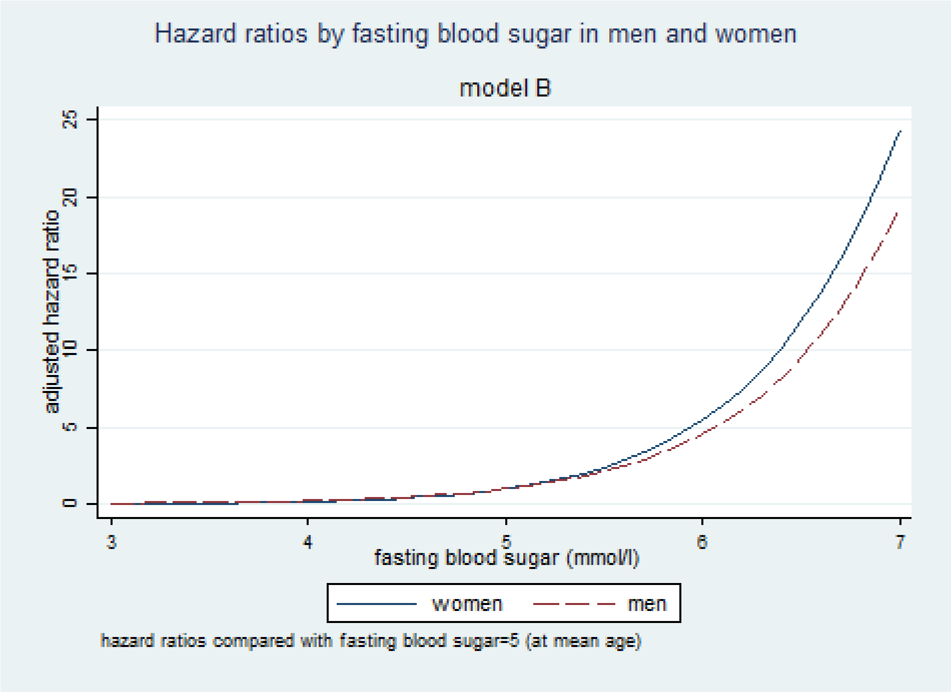

Supplementary Figure S1i

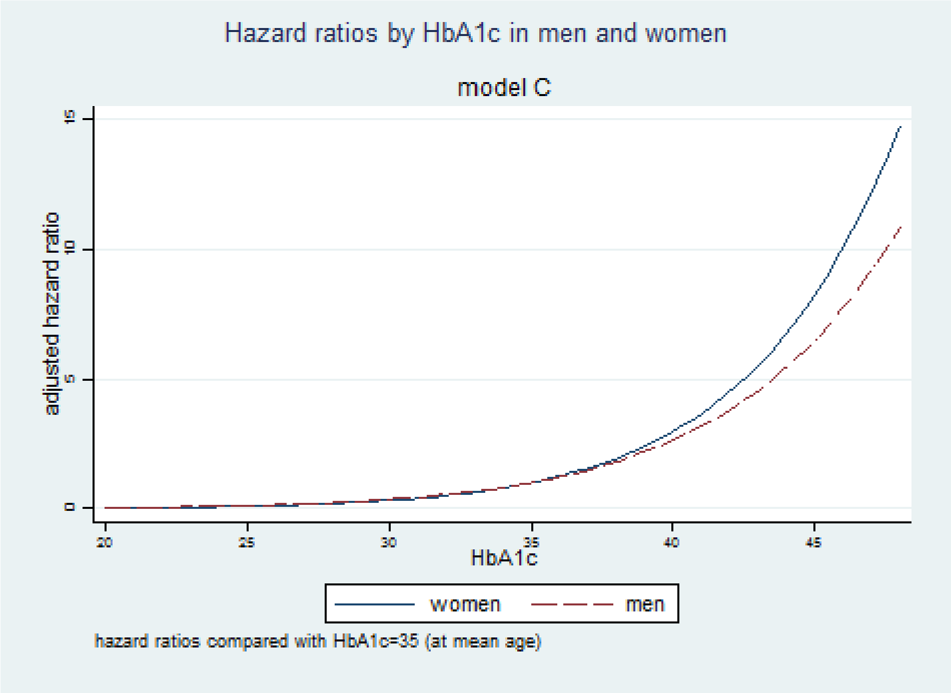

Supplementary Figure S1j

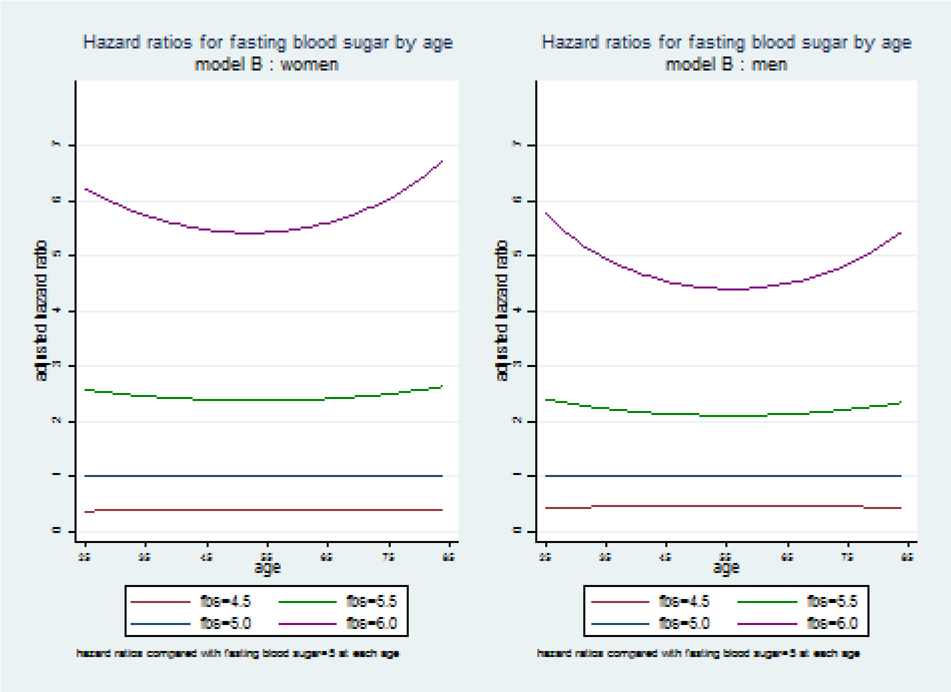

Supplementary Figure S1k

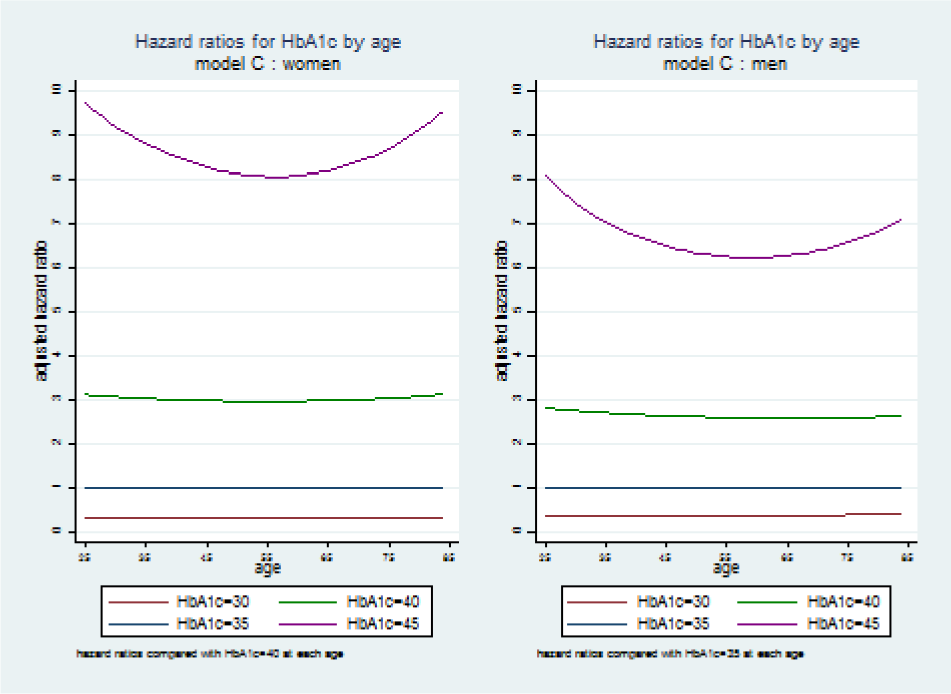

Supplementary Figure S2a and 2b

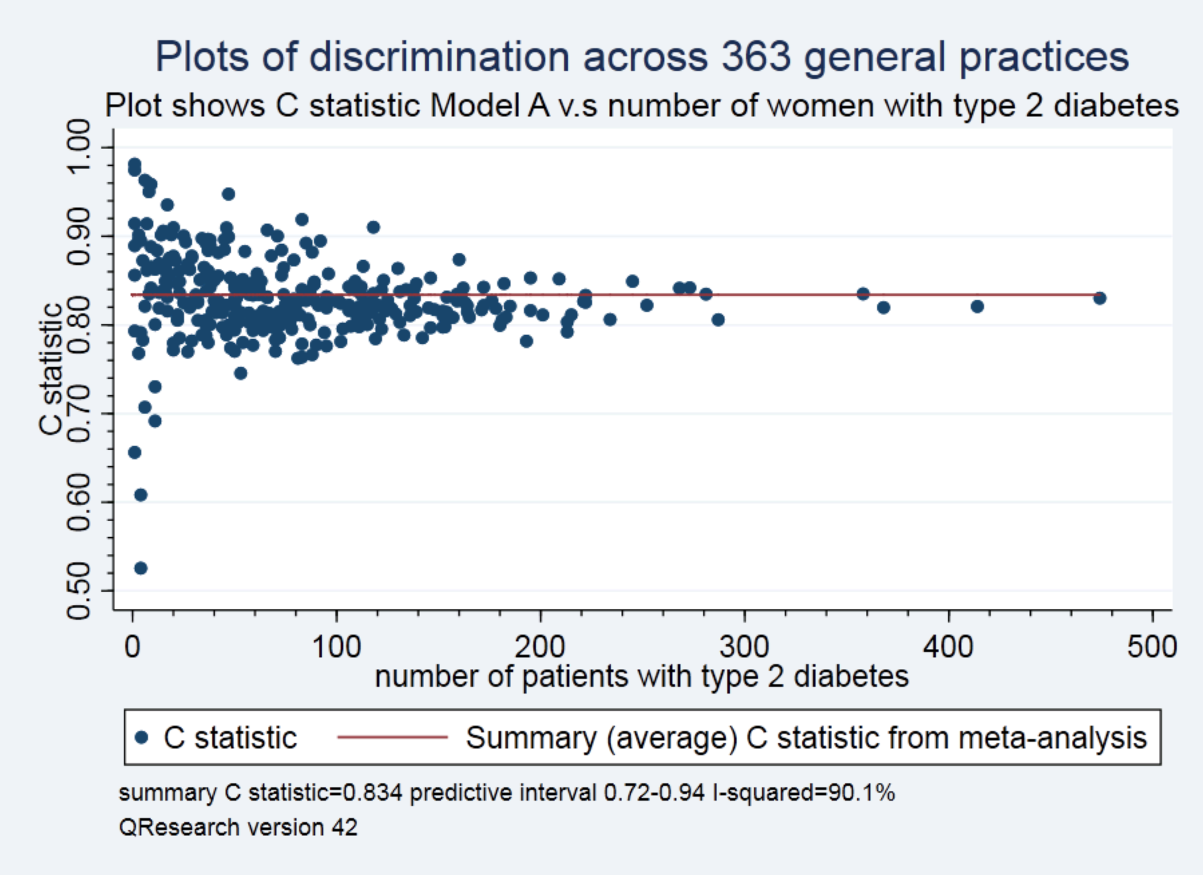

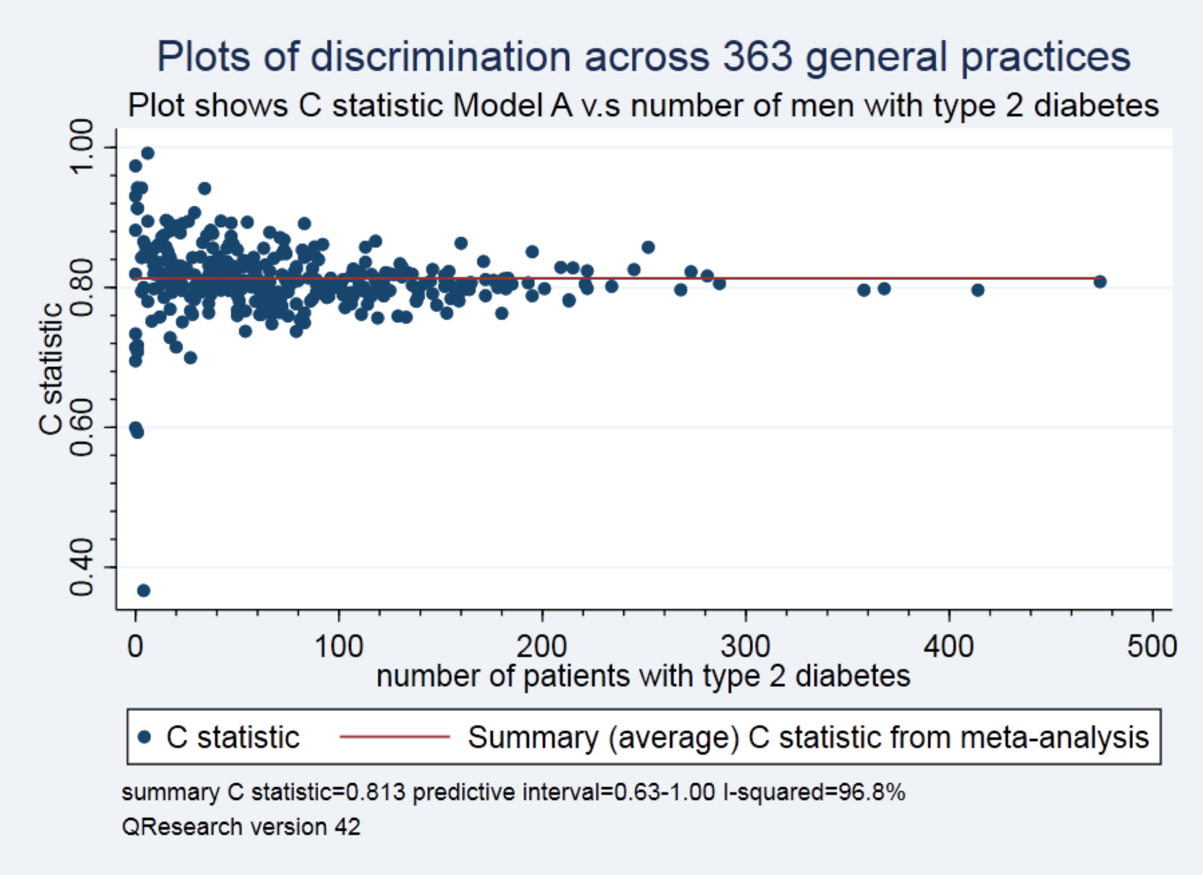

Supplementary Figure S2c and 2d

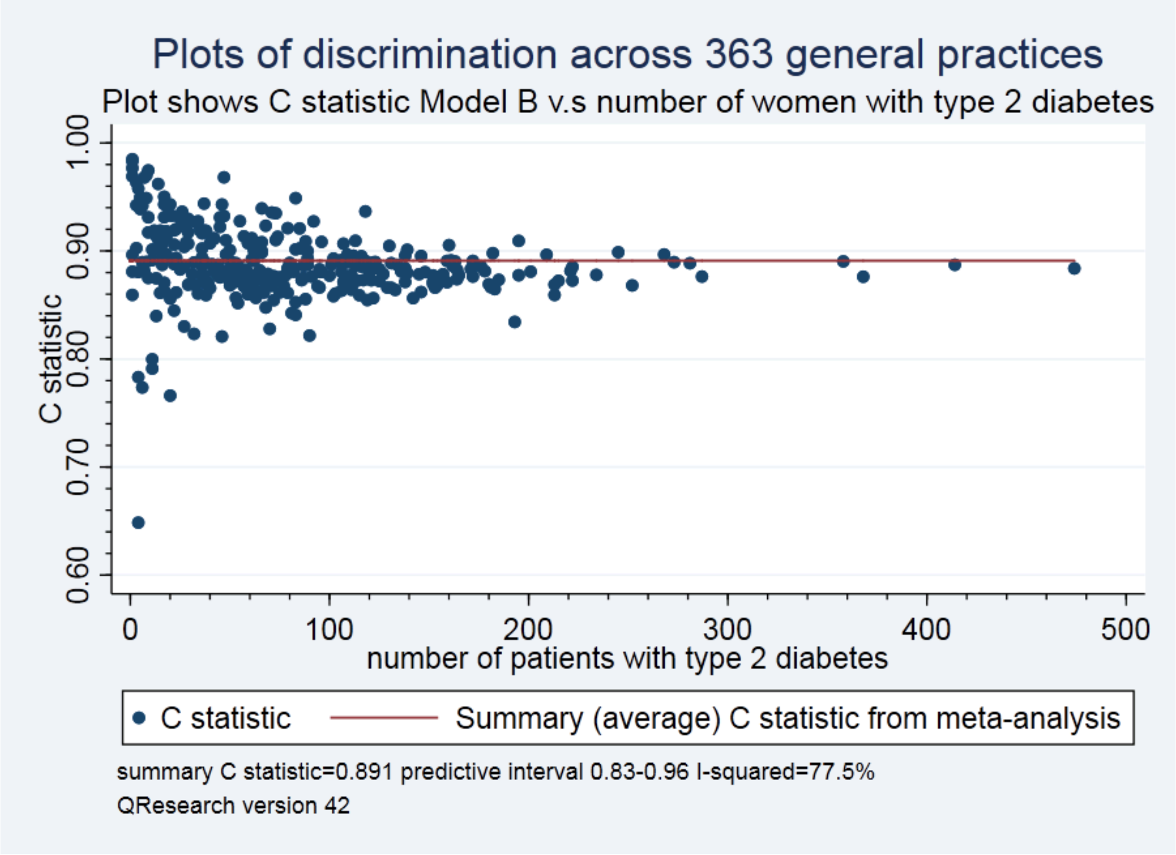

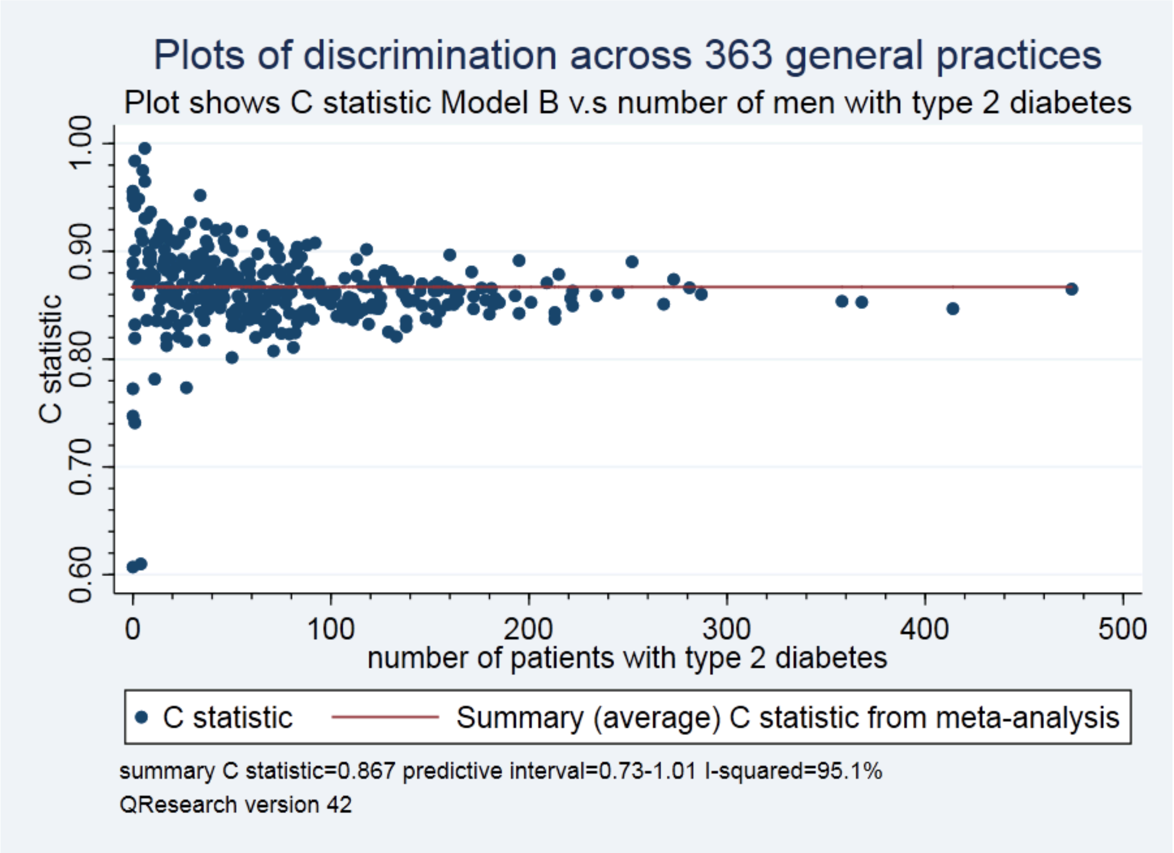

Supplement: Supplementary file 1 — Supplementary information: supplementary tables and figures [file hipj040627.ww1.pdf]
